# Ecosystem functioning of two marine food webs in the North‐Western Ionian Sea (Central Mediterranean Sea)

**DOI:** 10.1002/ece3.5527

**Published:** 2019-09-03

**Authors:** Pasquale Ricci, Simone Libralato, Francesca Capezzuto, Gianfranco D’Onghia, Porzia Maiorano, Letizia Sion, Angelo Tursi, Cosimo Solidoro, Roberto Carlucci

**Affiliations:** ^1^ Department of Biology University of Bari Aldo Moro Bari Italy; ^2^ CoNISMa Roma Italy; ^3^ Oceanography Division OGS (Istituto Nazionale di Oceanografia e di Geofisica Sperimentale) Trieste Italy

**Keywords:** benthic‐pelagic coupling, Ecopath model, functional traits, keystone species, trophic structure

## Abstract

The ecosystem functioning of two marine food webs covering the north‐eastern (Salento) and south‐western (Calabria) sectors of the North‐Western Ionian Sea (NWIS) (Central Mediterranean Sea) was investigated through a food‐web model. Data inputs covered a wide set of ecological information applied to 58 functional groups (FGs). The sum of consumption and the mean predation mortality rate were calculated for benthic, demersal, and pelagic subsystems indicating the predator and prey roles of the FGs. A complex system of energy and biomass exchanges characterized the investigated food webs indicating an important benthic‐pelagic coupling. In the food webs of both areas, the regulation of flows between the benthic‐pelagic coupling seems to occur through the benthopelagic shrimps and the small pelagics due to their wasp‐waist control role. Differences were observed concerning the top predators. Odontocetes play this keystone role in the Salento food web. Anglers, bathyal squids, and sharks assume this functional role in Calabria. The geomorphology and hydrography in the NWIS could affect the biomass and energy exchanges in this coupling. The higher flows of consumption of the benthic system observed in the Calabria food web could be influenced by a widespread presence of canyons along the continental edge which increase the benthic productivity. In contrast, the flows of consumption in the Salento food web seem to be driven by the planktonic productivity supporting the pelagic, benthopelagic, and demersal compartments. This condition could be favored by the large extension of the shelf break zone. The food‐web models realized for the NWIS represent ideal platforms for the development of analysis with dynamic simulations. The comparative analysis of the two food webs by means of the FGs and their functional traits allowed the general pattern of ecosystem structure and functioning in the NWIS to be identified, making it an interesting approach to investigate the marine ecosystem.

## Graphical abstract

The ecosystem functioning of two marine food webs covering the north‐eastern (Salento) and south‐western (Calabria) sectors of the North‐Western Ionian Sea (NWIS) (Central Mediterranean Sea) was investigated by means of a tropho‐dynamic model. The comparative analysis of the two food webs by means the functional groups and their functional traits allowed the general pattern of ecosystem structure and functioning in NWIS to be identified, making it an interesting approach to investigate the marine ecosystem.

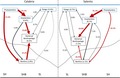

## 1 INTRODUCTION

The complexity of trophic structures in marine ecosystems has been extensively explored since the seminal study of Lindeman (1942) on the trophic‐dynamics of food webs. Such holistic analyses of cycles of mass, energy, and nutrients show how ecosystems respond to internal and external disturbances and recover from disturbance (Frank, Petrie, Fisher, & Leggett, 2011; Worm et al., 2006). The increasing global anthropogenic pressures on marine ecosystems (Halpern et al., 2008) require the study of the responses of marine food webs to such pressures, including the structural modifications in the species assemblages and the effects on the ecosystem functioning at different scales of organization (Cadotte, Carscadden, & Mirotchnick, 2011; Hooper et al., 2005). Induced changes to marine ecosystems might affect the roles assumed by each trophic group, the structural integrity and functioning of ecosystems and consequently their capacity to maintain ecosystem services (Worm et al., 2006). The contributions of species to ecological process are not always clear and many effects of the functional diversity on the ecosystem dynamics are little known (Bremner, 2008). As an example, few studies have investigated the effects of predator diversity in marine food webs, the role of omnivores in the cascading effects and more in general the effects of diversity at each trophic level (Bruno & O'Connor, 2005; Cardinale et al., 2006; Duffy et al., 2007). The main challenges derive from the complexity in defining a priori manipulations of functional traits to be implemented within empirical experiments and the difficulty of testing changes of functional diversity in a dynamic and holistic context such that of food webs (Lefcheck & Duffy, 2015). Within this context, modeling marine food webs seems to be an effective tool to highlight the role of species within the modeled ecosystem (functional niche, equivalence, redundancy, complementarity, etc.) (e.g., Mutshinda, Finkel, Widdicombe, & Irwin, 2016) and, by describing complex ecological structures, can help disentangle the effects of functional diversity on ecosystem processes (e.g., Holzwarth, Rüger, & Wirth, 2015). Modeling marine food webs has shown great potential for investigating the changes due to environmental variability and climate change (Heymans, Coll, Libralato, Morissette, & Christensen, 2014; Libralato, Caccin, & Pranovi, 2015), exploitation of fishing resources and aquaculture management (Forestal, Coll, Christensen, & Die, 2012; Libralato et al., 2010), as well as pollution, nutrient enrichment and the impact of alien species (Daskalov, 2002; Fulton, 2010; Pranovi et al., 2003).

The main objective of this study was to describe the ecosystem functioning of two marine food webs in the North‐Western Ionian Sea (Central Mediterranean Sea), identifying the species roles and setting the basis for an integrated approach in the area according the main goals of the EU Marine Directives (EU, 2008, 2013). The Common Fisheries Policy (CFP EU, 2013) worries about the loss of biodiversity and the need to study biodiversity in a holistic way to maintain marine habitats in a healthy, clean, productive, and resilient condition in order to achieve Good Environmental Status (GES) by 2020, as required by the EU Marine Strategy Framework Directive (EU, 2008). Within this context, the investigated area is characterized by great environmental variability (Carlucci et al., 2018 and references therein) and by the presence of different habitats (D'Onghia et al., 2012; 2016Calculli, Capezzuto, Carlucci, Carluccio, Grehan et al., 2016). Such variability is reflected in the diversity of both benthopelagic and demersal assemblages (Maiorano et al., 2010) as well as the spatial distribution of the fishing fleets that exploit the demersal resources (Russo et al., 2017). Some studies have explored the general structure of the demersal assemblages in the North‐Western Ionian Sea (Capezzuto et al., 2010; D'Onghia, Mastrototaro, Matarrese, Politou, & Mytilineou, 2003; D'Onghia, Tursi, Maiorano, Matarrese, & Panza, 1998; Maiorano et al., 2010) and the food web in the biological community of cold‐water corals within the same basin (Vassallo et al., 2017). However, a study focused on marine food webs and the ecological role of species has never been carried out in the area before. Therefore, two food‐web models were developed covering the north‐eastern and south‐western sectors of the North‐Western Ionian Sea to delineate the similarities and differences in structure and functioning of these marine food webs.

## 2 MATERIAL AND METHODS

### 2.1 Study area

**Figure 1:**
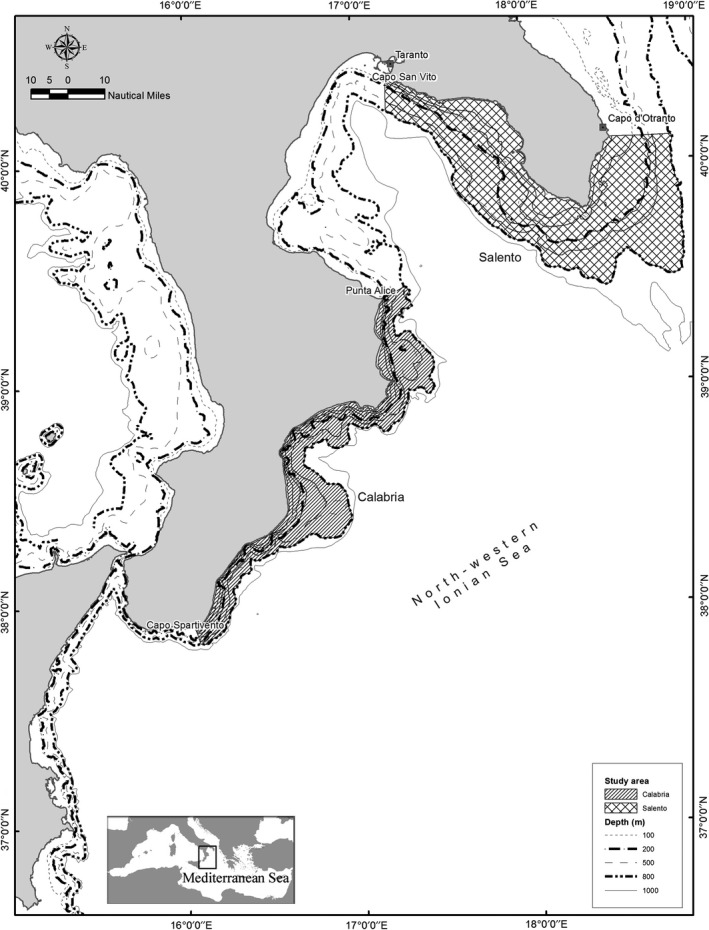
The two selected study areas for the implementation of food‐web models in the NWIS

The North‐Western Ionian Sea (NWIS), located in the Central Mediterranean Sea (Figure 1), is characterized by complex geomorphology and oceanography, as well as generally low productivity (Lazzari et al., 2012). The general hydrographic conditions determine substantial differences in salinity and temperature values within the NWIS area and the large‐scale circulation undergoes decadal changes resulting in local modification of physical, biogeochemical, and ecological conditions (Civitarese, Gacic, Lipizer, & Eusebi Borzelli, 2010). Within this region, two areas show distinctive characteristics and were used as domains for two distinctive food webs: the Salento area in the north‐eastern sector and the Calabrian area in the south‐western sector (Figure 1). The Salento sector is characterized by a broad continental shelf, rocky bottoms dominate on the shelf, and it is rich in marine caves of high ecological importance. On the contrary, in the Calabria sector, the shelf is generally narrow, and numerous submarine canyons are located along the coasts (Senatore, Mirabile, Pescatore, & Tramutoli, 1980).

The Salento food‐web model (SAL) represents the domain delimited by Capo Otranto and Capo San Vito (Taranto), which extends between 10–800 m in depth and covers an area of approximately 6,660 km^2^. The continental shelf (considered from 10 to 200 m depth) and slope (down to 800 m in depth) cover approximately 3,130 and 3,530 km^2^, respectively. The Salento area is characterized by the presence of many sensitive habitats, such as the Santa Maria di Leuca (SML) coral province, characterized by living *Madrepora‐Lophelia*‐bearing coral mounds, located between 350 and 1,100 m in depth (2016Calculli, Capezzuto, Carlucci, Carluccio, Maiorano et al., 2016; D'Onghia et al., 2012; 2016Calculli, Capezzuto, Carlucci, Carluccio, Grehan et al., 2016), and sea‐lily facies *Leptometra phalangium* on the shelf edge located in the eastern area. Furthermore, *Posidonia oceanica* and *Cymodocea nodosa* meadows and coralligenous biocenosis are widespread in this geographic sector (Figure 1).

The domain of the Calabrian food‐web model (CAL) extends from Punta Alice to Capo Spartivento for approximately 3,469 km^2^ in the depth range 10–800 m (Figure 1). The continental shelf covers approximately 965 km^2^ while the slope (down to 800 m in depth) covers approximately 2,504 km^2^. This area is characterized by the presence of sensitive habitats, such as *C. nodosa* meadows, by a restricted shelf edge and especially by the presence of many submarine canyons.

### 2.2 The Ecopath modeling approach

The Ecopath within the Ecosim (EwE) modeling approach (http://www.ecopath.org; Christensen, Walters, Pauly, & Forrest, 2008) was used to develop the two food‐web models for the North‐Western Ionian Sea marine system. The EwE approach has been developed over the last 30 years, and it is now widely used for building and analyzing mass balance food‐web models of aquatic and terrestrial ecosystems worldwide (Christensen & Walters, 2004; Colléter et al., 2015). EwE allows the static (e.g., Agnetta et al., 2019), time dynamic (e.g., Celić et al., 2018), and spatio‐temporal dynamic (e.g., Coll, Steenbeek, Sole, Palomera, & Christensen, 2016; Walters, 2000) approaches to be implemented through the Ecopath, Ecosim, and Ecospace modules, respectively. Food webs are described by means of Functional Groups (FGs), each representing a species, a life stage of a species, or a group of species with similar trophic, ecological, and physiological features. Links between FGs are formally described by a set of linear equations (Christensen et al., 2008). The state variable initial conditions are the biomasses of the groups i and the parameters include the production rate (P/B_i_), consumption rate (Q/B_i_), diet composition (DC_ij_), unassimilated food (U/Q_i_), catches (Y_i_), and exports (E_i_) for each group. The system of equations is solved by providing EwE with information on three out of the four basic parameters B_i_, (P/B_i_), (Q/B_i_) and EE_i_. Further details on the EwE modeling approach can be found in review literature (Christensen & Walters, 2004; Christensen et al., 2008; Heymans et al., 2016).

### 2.3 Model structure: definition of functional groups

The definition of FGs in food‐web models is usually based on cluster analysis of quantitative diet information, somewhat guided by taxonomic features, life‐history traits, and expert judgment (Banaru et al., 2013; Coll, Santojanni, Palomera, Tudela, & Arneri, 2007; Moutopoulos, Libralato, Solidoro, & Stergiou, 2013; Tsagarakis et al., 2010). However, these aggregation criteria of species often do not support the ecological reality of the group. In this study, we defined the functional groups by combining the trophic similarity and the bathymetric distribution of species, considering these as the main driving‐forces in the definition of groups for the benthopelagic and demersal species sampled by the “MEDiterranean International Trawl Survey” (MEDITS time series 1995–2012) (Table S1). This choice was based on the ecological assumption of foraging arena theory (Ahrens, Walters, & Christensen, 2012), where a consumer is influenced by the availability of resources in the area where it lives.

A total of 276 species belonging to the demersal and benthopelagic assemblages were aggregated within 39 FGs selected by an original reiterative aggregation method, according to the similarity in qualitative diet information (also considering information from http://www.FishBase.org) and in the bathymetric distribution of species. One bathymetric distribution indicator of the species biomass was the Centre Of Gravity (COG, Daget, 1976), a synthetic measurement that indicates the depth to which a species shows the highest biomass concentration, with a value of variance indicating the displacement of species biomass between bathymetric layers, and it is expressed as follows:

COG=(X_1+2X_2+3X_3+4X_4+…+nX_n)/∑X_i

where *X* represents the value of the average biomass of the species in layer i. In particular, 8 bathymetric layers of 100 m were identified between 10 and 800 m in depth. The quantitative information on diet preferences (in weight) was collected for 129 species out of a total of 276 species sampled in the benthopelagic and demersal assemblages of study areas, using the published scientific and gray literature both of local and nearby geographical areas. The analysis of diet data was carried out by implementing a bi‐clustering on the matrix of prey‐predator relationships, also using the vector of weighting factor [COG], implemented by means of a Microsoft Visual Basic routine (Appendix S1).

The remaining 147 species lacking in diet information were successively grouped according to their life‐history traits, information from qualitative diets, and habitat preferences identified by a self‐organizing map analysis (Carlucci et al., 2018). However, due to their ecological and commercial importance, several species were ungrouped and defined as a single FG: the blackmouth catshark (*Galeus melastomus*), the hake (*Merluccius merluccius*), the red mullet (*Mullus barbatus*), the anglers (*Lophius piscatorius* and *L. budegassa*), the bluntsnout grenadier (*Nezumia sclerorhynchus*), the red giant shrimp and blue and red shrimp (*Aristaeomorpha foliacea* and *Aristeus antennatus*, respectively), the deep‐water rose shrimp (*Parapaeneus longirostris*), and the golden shrimp (*Plesionika martia*). Moreover, other components of the NWIS ecosystem, such as the plankton community, the benthic invertebrates, the benthic producers, the top predators, mammals, seabirds, the sea turtles, and three non‐living groups namely bottom detritus, dead discards, and suspended organic matter (Detritus, Discard and Marine snow, respectively; see also Agnetta et al., 2019) were described in 19 additional FGs (Table 1).

**Table 1:** List of functional groups with the corresponding short FG name, domain, and bathymetric layer used in the food‐web models

*N*.	Functional GROUP	Short FG name	Domain, Bat. layer	*N*.	Functional GROUP	Short FG name	Domain, Bat. layer
1	Odontocetes	Odontocetes	P	30	Bluntsnout grenadier	Blunt grenad	B‐D SL
2	Fin Whale	F whale	P	31	Slope Squids benthopelagic feeders	SL_Squids_BP	BP SL
3	Loggerhead Turtle	Log turtle	P	32	Shelf Break‐Slope Squids benthopelagic feeders	SHB_Squids_BP	BP SHB
4	Seabirds	Seabirds	P	33	Shelf‐Shelf Break Cephalopods benthopelagic feeders	SH_Ceph_BP	BP SH
5	Large pelagic fishes	L pelagics	P	34	Slope Octopus and Bobtail Squids benthic feeders	SL_Octopus_bent	B‐D SL
6	Slope Sharks and Rays benthic feeders	SL_SharkRays_bent	B‐D SL	35	Shelf Break‐Slope Bobtail Squids benthopelagic feeders	SHB_BSquids_BP	BP SHB
7	Shelf‐Shelf Break Sharks and Rays benthopelagic feeders	SH‐SHB_SharkRays_BP	BP SHB	36	Benthopelagic Shrimps	Shrimps BP	BP SL
8	Shelf Sharks and Rays benthic feeders	SH_SharkRays_bent	B‐D SH	37	Slope Decapods Scavengers.	SL_Decap_Scav	B‐D SL
9	Slope Sharks benthopelagic feeders	SL_Sharks_BP	BP SL	38	Shelf Break‐Slope Crabs.	SL_Crabs	B‐D SL
10	Blackmouth catshark	B catshark	B‐D SL	39	Shelf‐Shelf Break Crabs	SHB_Crabs	B‐D SH
11	Slope Demersal fishes opportunistic feeders	SL_DemF_opp	B‐D SL	40	Shelf Crabs	SH_Crabs	B‐D SH
12	Shelf Break‐Slope Demersal fishes generalist feeders	SHB‐SL_DemF_gen feed	B‐D SHB	41	Deep‐Water Rose shrimp	DWR shrimp	B‐D SHB
13	Shelf‐Shelf Break Demersal fishes generalist feeders	SH‐SHB_DemF_gen feed	B‐D SH	42	Red Giant shrimp	RG shrimp	B‐D SL
14	Shelf‐Shelf Break Demersal fish piscivorous	SH‐SHB_DemF_pisc	B‐D SH	43	Red and Blue shrimp	RB shrimp	B‐D SL
15	Slope Bathypelagic fishes piscivorous	SL_BathypelF_pisc	BP SL	44	Golden shrimp	G shrimp	B‐D SL
16	Slope Demersal fishes shrimps feeders	SL_DemF_shrimps feed	B‐D SL	45	Polychaetes	Polychaetes	B‐D
17	Slope Fishes benthopelagic crustaceans feeders	SL_F_BP crust feed	BP SL	46	Macrobenthic invertebrates	Macrobent inv	B‐D
18	Shelf Break‐Slope Fishes benthopelagic crustaceans feeders	SHB_F_BP crust feed	BP SHB	47	Gelatinus plankton	Gel plank	P
19	Shelf‐Shelf Break Demersal fishes benthic crustaceans feeders	SH_DemF_bent crust feed	B‐D SH	48	Suprabenthic crustaceans	Supbent crust	B‐D
20	Shelf‐Shelf Break Demersal fishes benthic invertebrate feeders	SH_DemF_bent inv feed	B‐D SH	49	Macrozooplankton	Macrozooplank	P
21	Slope Fishes zooplanktivorous	SL_F_planktivorous	BP SL	50	Mesozooplankton	Mesozooplank	P
22	Shelf Break Fishes zooplanktivorous	SHB_F_planktivorous	BP SHB	51	Microzooplankton	Microzooplank	P
23	Small pelagic fishes	S pelagics	P SH	52	Bacterioplankton	Bact plank	P
24	Medium pelagic fishes	M pelagics	P SH	53	Seagrasses and Microphytobenthos	Seagrasses‐algae	B‐D
25	Macrourids	Macrourids	B‐D SL	54	Large phytoplankton	L phytoplank	P
26	Myctophids	Myctophids	BP SHB	55	Small phytoplankton	S phyotplank	P
27	Red mullet	R mullet	B‐D SH	56	Marine snow	MS	
28	Hake	Hake	B‐D SHB	57	Discards	Disc	
29	Anglers	Anglers	B‐D SHB	58	Detritus	Det	

Note

Pelagic (P), Benthopelagic (BP), Benthic‐Demersal (B‐D), Shelf (SH), Shelf Break (SHB), and Slope (SL).

Both food‐web models were composed of 58 FGs (the respective names of which were based on the bathymetric layer, the taxonomy and the characteristic feeding habits of the group (Table 1 and Table S1).

### 2.4 Model parametrization: input data from 1995 to 1997

The input parameters and data sources for Biomass (as t/km^2^), annual production and consumption rates (P/B and Q/B), annual fishery landings and discards are reported in Table S2. The diet composition matrices and data sources of FGs are reported in (Figure S1–S2). The Ecotrophic Efficiency (EE) was fixed for the polychaeta, macrobenthic invertebrate groups at a value of 0.90, gelatinous plankton, and suprabenthic crustacean groups at a value of 0.95 and for the macro and mesozooplankton FGs at a value of 0.99 (Table S2) (Heymans et al., 2016). The biomass data from the MEDITS trawl surveys do not account for the catchability of the fishing gear, and thus, data were corrected using a catchability factor by species (qi) obtained from the literature whenever possible (Fiorentino et al., 2013; Fraser, Greenstreet, & Piet, 2007). In some instances, catchability by species for demersal species was evaluated by comparison of MEDITS estimates with other data (e.g., benthic samples, other fishing gears, stock assessments) in order to determine more accurate absolute densities at sea: although this implies great uncertainty, it is a necessary step which is not always explicit in EwE modeling (see for example Arreguin‐Sanchez, 1996).Biomass data of Odontocetes, Fin whale, and Loggerhead turtle were estimated by abundance data (N/km) obtained from the OBIS SeaMap (Halpin et al., 2009) and the mean individual weight used in other models (Piroddi, Bearzi, & Christensen, 2010).

Official fishery landings by species were provided from the Fisheries and Aquaculture Economic Research for the Ministry of Agricultural Food and Forestry Policies (MIPAAF). Data referring to annual commercial landings for the period 2006–2015 were processed in order to reconstruct disaggregated landings for trawls, long lines, nets, other gears, and purse seines in the period 1995–2005. Discards of the trawl fishery by species with commercial value (undersize individuals) were calculated using the discard rate estimated by STECF (2004). The discard fraction for species or functional groups of no commercial value was calculated on the basis of the proportion of commercial and no commercial discard in MEDITS data and local references (D'Onghia, Carlucci, Maiorano, & Panza, 2003). Discard for others gears was obtained from the scientific literature relative to neighboring Mediterranean areas (Goncàlves et al., 2007; Morello, Froglia, Atkinson, & Moore, 2005; Tsagarakis, Palialexis, & Vassilopoulou, 2014; Voultsiadou, Fryganiotis, Porra, Damianidis, & Chintiroglou, 2011) eventually correcting discard rate on the basis of the knowledge and experience of local fishermen. Only the official landing and discard data were used in the analysis, while estimates related to illegal, unreported, and unregulated (IUU) and recreational fishery catches for the Ionian area (Piroddi et al., 2015) were not considered for due to their high uncertainty level. In particular, the differences between the Ionian area considered in Piroddi et al. (2015) and our model areas represent a critical issue in the disaggregation of species, gears and for correct evaluation of the IUU and recreational fishery catches. Nevertheless, the estimates reported in Piroddi et al. (2015) represented negligible contributions to the official landing data and discards used in NWIS models, where our landing and discard data were two orders of magnitude larger than the IUU and recreational fishery catches. Thus, although the fishery data adopted in our study were slightly underestimated, the validity and the robustness of the data were very high.

The models were constructed considering data for a reference period (1995–1997) that was chosen to facilitate future successive steps of time‐dynamic model analysis by means of the Ecosim routine (Christensen et al., 2008).

### 2.5 PREBAL analysis and balancing steps

The pre‐balancing analysis (PREBAL, Link, 2010) was carried out to assess the coherence of the input data with the basic thermodynamic laws, rules, and principles of ecosystem ecology at the system level (Alexander et al., 2015; Heymans et al., 2016) (see Figure S3–S4).

Initially, the NWIS models were not balanced mostly due to of several EE values higher than 1 in different FGs. Therefore, the models were manually balanced adopting a top–down approach (Mackinson & Daskalov, 2007) consisting of slight modifications to the production rates within the range by species.

When the Pedigree Index (see Section 2.62.6) indicated less reliable values in the DC matrix, the input data P/B and Q/B were changed during the balancing steps. The biomass values from trawl surveys were modified exclusively when the catchability data were lacking or highly uncertain, and always within the observed range.

Net food conversion efficiencies (P/Q [0.05–0.3]), respiration/assimilation (R/A [<1]), and production/respiration (P/R [<1]) ratios were checked to be within expected limits (Christensen et al., 2008).

Cannibalism causes several problems in the model balancing (Heymans et al., 2016). Therefore, it was decreased for hake, sharks, demersal fish groups, squids, shrimps, and crabs groups.

### 2.6 Ecological indicators estimated by the model

The “pedigree” of each input data was defined, on the basis of the source of data and its accuracy (whether it was taken from a model or original field sampling, from the studied system or from a similar system) and these values were used to assess the overall quality of the model in the form of a pedigree index that varies between 0 (low quality) and 1 (high quality). Ecological indices were used to analyze the role of species and ecosystem structure based on trophic flow analysis, thermodynamic concepts and network theory (Christensen & Walters, 2004). In particular, the fractional Trophic Level (TL), Ecotrophic Efficiency (EE), Omnivory Index (OI), and Keystoness Index (KSi) were calculated for each FG comparing the values obtained in Calabria and Salento.

The TL of each FG was calculated as follows:

$${\mathrm{TL}}_{i}=1+\sum {\mathrm{DC}}_{\mathrm{ij}}\cdot {\mathrm{TL}}_{i}$$

where j is the predator of prey i, DC_ji_ is the fraction of prey i in the diet of predator j and TL_i_ is the trophic level of prey i, and conventionally assuming a TL of 1 for primary producers and detritus. This summarizes the ecological position of each FG within the food web.

The OI was calculated for each FG as the variance of the trophic level of a consumer's prey groups (Pauly, Soriano‐Bartz, & Palomares, 1993). When the OI value is zero, the consumer in question is specialized (i.e., it feeds on a single trophic level). A large value indicates that the consumer feeds on many trophic levels (see Libralato, 2013).

The KSi was calculated through Mixed Trophic Impact analysis (MTI, Ulanowicz & Puccia, 1990), which quantifies direct and indirect trophic interactions between functional groups i and j through the elements of the MTI matrix (*m*_ij_). According to Libralato, Christensen, and Pauly (2006), the overall effect (*ε*_i_) of functional group i is estimated as:

$$\varepsilon_{i}=\sqrt{\sum_{j=1}^{n}m_{\mathrm{ij}}^{2}}$$

where the impact on the group itself (*m*_ij_ with i = j) is not considered, and *ε_i_* is calculated as a relative value with respect to the maximum (see also Libralato et al., 2006). The KS_i_ is expressed as:

$${\mathrm{KS}}_{i}=\log\left[\varepsilon_{i}\left(1-p_{i}\right)\right]$$

where p_i_ is the relative biomass of the group, excluding detritus biomass. The indicated keystone and dominant groups are system specific (Coll, Santojanni, Palomera, & Arneri, 2009; Libralato et al., 2006) but previous analyses showed that the keystone proportion is also sensitive to perturbations (Coll & Libralato, 2012; Heymans et al., 2014).

The association of each FG to the pelagic (P), benthopelagic (BP), and benthic‐demersal (B‐D) subsystems allowed investigation of the structure and relationships between the functional groups belonging to different compartments (Table 1). Accordingly, indicators were also calculated for the demersal community (39 FGs) aggregated into three bathymetric layers: Shelf (SH), Shelf Break (SHB), and Slope (SL).

The sum of consumption (t km^−2^ year^−1^) and the mean predation mortality rate were calculated as a percentage for each subsystem (P. BP and B‐D), separating the groups into predators and prey (Hattab et al., 2013) and for the 39 FGs of demersal and benthopelagic assemblages aggregated into SH, SHB and SL.

## 3 RESULTS

**Figure 2:**
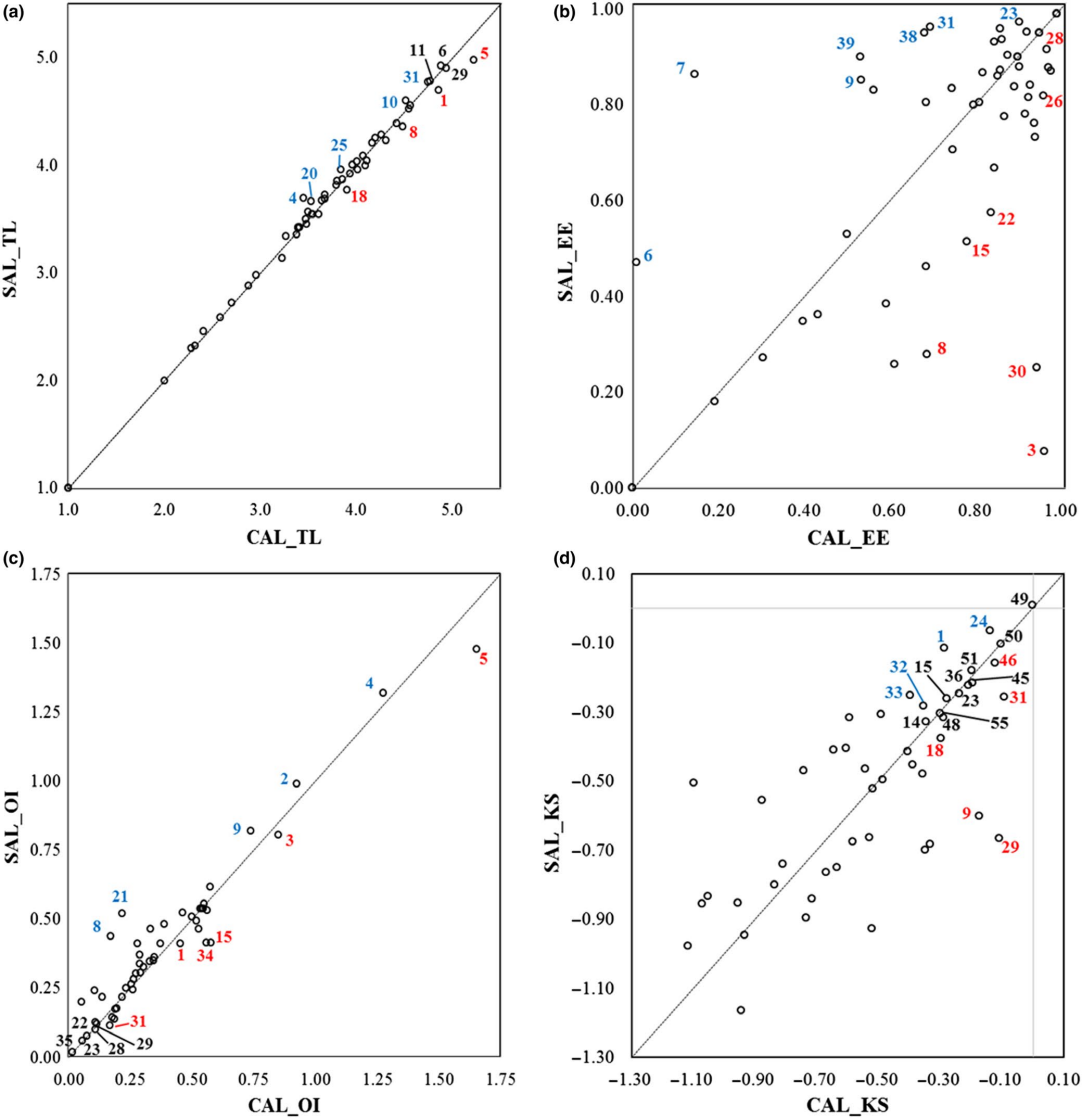
(a–d) Comparison of food‐web indicators between the CAL and SAL models (on the y and x axes, respectively). Numbers refer to FG codes. Those FGs showing relatively equal values in both food webs are black‐colored; FGs with a higher value for a food‐web indicator in one of the two webs are either red (Calabria) or blue‐colored (Salento). The codes of indicators correspond to: Trophic Level (TL), Ecotrophic Efficiency (EE), Omnivory Index (OI), and Keystoness Index (KSi)

The pedigree index for both CAL and SAL models was 0.68. The main ecological indices of the SAL and CAL models are compared in Figure 2 (Table S3). The TLs were substantially similar for most of the functional groups' in the two models. The highest TL values were estimated for the following groups: L pelagics, Odontocetes, SL_SharkRays_bent, the Anglers, and SL_DemF_opp (FG 5, 1, 6, 29, and 11, respectively) (Figure 2a). L pelagics, Odontocetes together with SH_SharkRays_bent and SHB_F_BP crust feed (FGs 8 and 18, respectively) showed higher TL in the Calabrian food web. In contrast, the TLs of SL_Squids_BP, B catshark, Macrourids, SH_DemF_bent inv feed and Seabirds (FGs 31, 10, 25, 20 and 4, respectively) were higher in SAL than in CAL.

Ecotrophic efficiency estimates showed high similarity between the two models for a set of FGs (1, 2, 4, 19, 27, 12, 23, 28, 34, 35, 40, and 41) (Figure 2b), but SH‐SHB_SharkRays_BP, SL_SharkRays_bent, SL_Sharks_BP, SHB_Crabs, SL_Crabs, and SL_Squids_BP (FGs 7, 6 9, 39, 38, and 31, respectively) had a much higher EE in the Salento than in the Calabrian food web. Conversely, higher EE values were estimated in CAL for the following FGs: Log turtle, Blunt grenad, SH_SharkRays_bent, SL_BathypelF_pisc, SHB_F_planktivorous, and Myctophids (FG 3, 30, 8, 15, 22, and 26, respectively).

The estimated OI showed that in both food webs the most generalist groups are L pelagics, Seabirds, F whale, Log turtle, and SL_Sharks_BP (FG 5, 4, 2, 3, and 9, respectively) (Figure 2c and Table S3). Low values of OI were found for specialized consumers, such as the Anglers, Hake, SL_Squids_BP, SHB_BSquids_BP, SHB_F_planktivorous, and S pelagics (FGs 29, 28, 31, 35, 22, and 23, respectively). The Odontocetes, SL_BathypelF, and SL_Octopus_bent (FGs 1, 15, and 34, respectively) showed higher values of OI in CAL than in SAL, differently from SL_F_planktivorous and SH_SharkRays_bent (FG 21 and 8, respectively).

The KSi rank the functional groups within the investigated food webs in terms of the key roles they play (Figure 2d). The main keystone group was Macrozooplankton (FG 49) in both food webs followed by the Meso‐Microzooplankton groups, Polychaetes, Shrimps BP, S pelagics (FGs 50, 51, 45, 36 and 23, respectively). The Odontocetes, Medium pelagic fishes, SHB_Squids_BP, and SH_Ceph_BP (FGs 1, 24, 32 and 33, respectively) were the main keystone groups in the Salento food web. Higher values of KSi were estimated for Macrobent inv, SL_Squids_BP, Anglers, SL_Sharks_BP and SHB_F_BP crust feed (FGs, 46, 31, 29, 9, and 18, respectively) in the Calabrian food web. Similar values of KSi were observed in both food webs for SL_BathypelF_pisc, S phytoplank, Supbent crust, and SH‐SHB_DemF_pisc (FGs 15, 55, 48 and 14, respectively).

**Figure 3:**
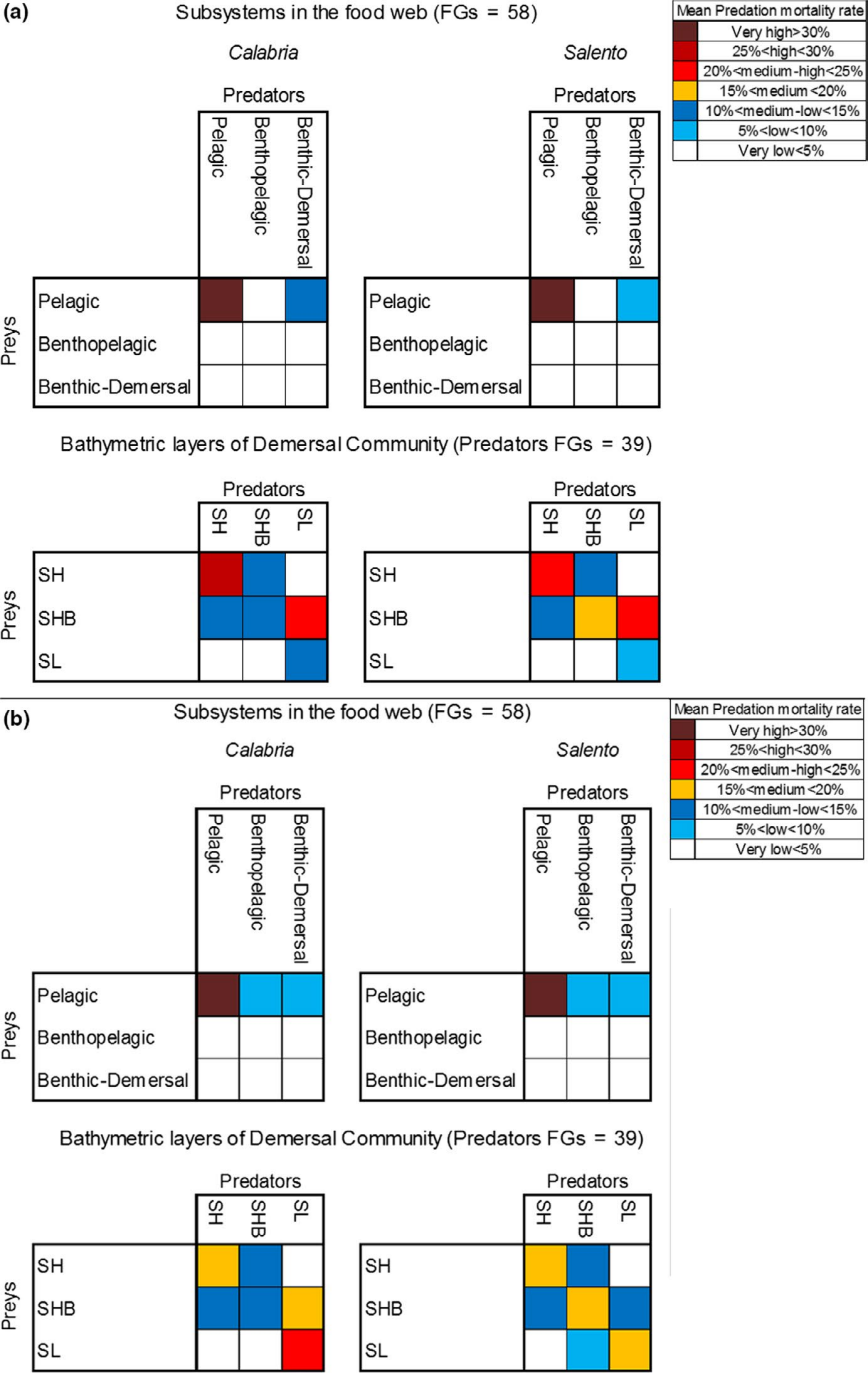
(a) Sum of Consumption flows (t km^−2^ year^–1^) and (b) Mean Predation mortality rate (year–1) between the FGs aggregated in the three sub‐systems (Pelagic, Benthopelagic, and Benthic‐Demersal) and three depth layers (Shelf SH, Shelf Break SHB, and Slope SL) of the Calabria and Salento food webs

The sum of consumption estimated for both trophic webs did not show differences within the pelagic (P), Benthopelagic (BP), and Bentho‐demersal (B‐D) subsystems (Figure 3a upper part). The highest percentages of consumption flow were estimated for the consumers within the P subsystem (>30%). Higher percentage values of consumption flow were estimated for the consumers of B‐D compartment feeding on pelagic preys in the CAL (15%–20%) than in the SAL (5%–10%) model. The consumption flows were clearly different between the depth layers (Figure 3a lower part). The shallower consumers feeding on prey belonging to the SH layer showed higher values in the CAL (25%–30%) than in the SAL (<25%) model. On the contrary, the SHB consumers feeding on preys belonging to the SHB layer showed higher values in the SAL (15%–20%) than in the CAL (<15%) model. Whereas, the bathyal predators consuming preys located in the SL layer showed higher percentage values in the CAL (10%–15%) than in the SAL (>10%) model.

In both food webs, the highest values of mean predation mortality rate were estimated for the consumers of all compartments exploiting the pelagic preys (Figure 3b upper part). The higher percentage of predation mortality rate was observed for P consumers on the P preys in both food webs. Differences were observed for the bathyal predators feeding on prey distributed on the SHB and SL layers (Figure 3b lower part). Higher percentage values of mean predation mortality rates were estimated in the CAL than in the SAL food web for the SL predators exploiting the preys on the SHB and SL. On the contrary, the only higher percentage value recorded in SAL than in the CAL model was estimated for the SHB consumers feeding on the preys of SHB layer.

**Figure 4:**
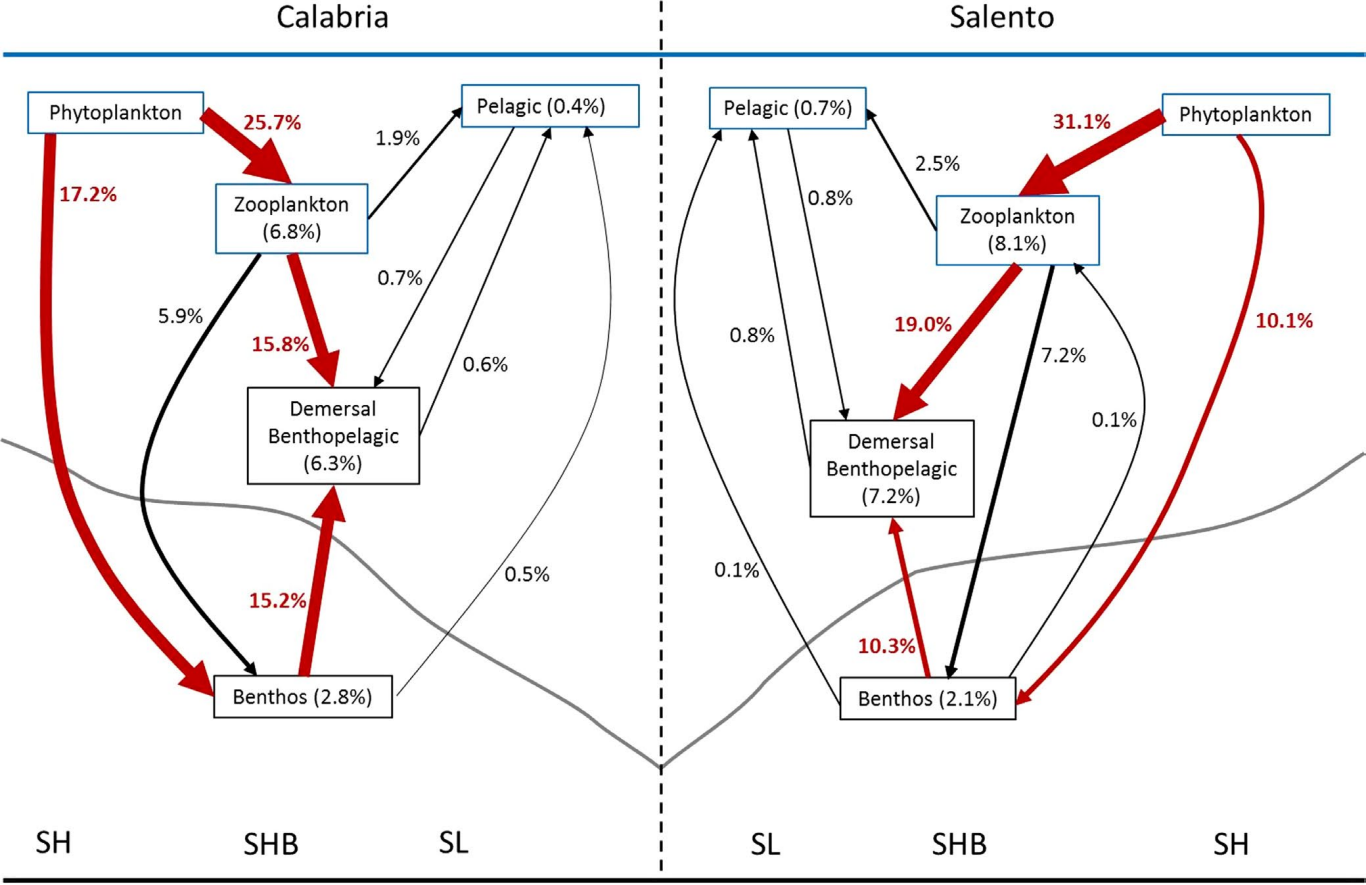
Representation flows of biomass (expressed as percentages) between planktonic, pelagic, demersal and benthic compartments and in the depth layers (Shelf SH, Shelf Break SHB, and Slope SL) of the Calabria and Salento food webs. The Plankton compartment is divided into phyto‐ and zooplankton. Flows to Detritus, Discard and Marine snow as well as flows of Bacterioplankton, Micro‐Mesozooplankton groups were not considered. The percentage within each box indicates the biomasses flows consumed by FGs belonging to the compartment. The thickness of the arrows is proportional to the magnitude of the input flows within each food web. Red color marks differences in the consumption flows between NWIS food‐web models. Gray curves indicate the bottom profile

The flows of the consumption between the pelagic, demersal, and benthic compartments differed between the two food webs, mostly due to the flows directed toward the demersal benthopelagic groups and zooplankton groups (Figure 4). Flows from the phytoplankton (FGs 54 and 55, respectively) to the zooplankton (FGs 47, 48, and 49, respectively) had higher estimated values in the SAL (31.1%) than in the CAL (25.7%) model. Differently, the flows from the benthos (FGs 45, 46, and 53, respectively) to the demersal benthopelagic compartment were higher in the CAL (15.2%) than in the SAL (10.3%) food web. Conversely, the groups of demersal fishes, crustaceans, and cephalopods consumed the macrozooplankton, gelatinous plankton and suprabenthic crustaceans (FGs 49, 47 and 48, respectively) more in the SAL (19.0%) than in the CAL (15.8%). The benthos groups consumed more phytoplankton in the CAL (17.2%) than in the SAL (10.1%).

## 4 DISCUSSION

The Salento and Calabrian food webs were modeled using detailed information on demersal and benthopelagic assemblages. The long‐term series of data (MEDITS 1995–2012), collected in the framework of a standardized survey investigating demersal and benthopelagic species (Bertrand, Gil de Sola, Papaconstantinou, Relini, & Souplet, 2000), ensured robustness in the description of the overall functional traits of the groups. In fact, the pedigree index for both the CAL and SAL models is higher than the values observed in 50 models provided by Morissette (2007). In particular, only five models show values higher than 0.60. Both NWIS trophic webs are implemented using a very high biological articulation (i.e., a relatively high number of functional groups; 58 FGs), and the overall model quality shows higher values than those calculated for other Mediterranean areas (Corrales et al., 2015). However, some information gaps were detected for low trophic level groups, such as suprabenthic crustaceans, polychaetes, macrobenthic invertebrates, and gelatinous plankton, which should be addressed by future research goals in the area. Despite the common model structure for the two food webs, some differences emerge as a consequence of differences in geomorphological and hydrographic conditions influencing occurrence, distribution, and abundance of marine species in the NWIS (Carlucci et al., 2018).

### 4.1 Ecological roles and trophic interactions in the NWIS food webs

The trophic levels estimated for each functional group and the mean values calculated for each investigated food web (mean TL values of 3.5 in both food webs) are consistent with those reported for demersal and benthopelagic species in the Mediterranean Sea (Stergiou & Karpouzi, 2002).

The TL observed for Odontocetes in the Salento trophic web was lower than in Calabria. This could be explained by the fact that the common bottlenose dolphin, *Tursiops truncatus*, finds more suitable habitats in Salento due to the larger extension of the shelf and shelf break zones. Consequently, since *T. truncatus* is an opportunistic feeder, in Salento its consumption of discards is higher due to its foraging behind the fishing vessels (Carlucci et al., 2016). The lower trophic level of the Odontocetes group in the Salento food web also results from the convention that the discard TL is set to 1 as non‐living matter, which is an assumption that deserves in greater attention in future.

The highest values of EE indicated the Small pelagics as important resources for several consumers within the investigated food webs, confirming the role reported for the same groups in other Mediterranean models (Coll, Palomera, Tudela, & Sardà, 2006; Coll et al., 2007; Corrales et al., 2015; Tsagarakis et al., 2010). The EE values estimated for the sharks and rays distributed in shelf‐break and slope grounds (FGs 7, 6 and 9) were lower in the CAL than the SAL trophic web. This result could be due to lower vulnerability of sharks and rays in Calabria, where the presence of deep submarine canyons distributed along the continental margin provide natural refuges for these species, thus reducing their fishing mortality (D'Onghia et al., 2015; Fernandez‐Arcaya et al., 2017; Sion et al., 2019). Differently, the higher presence of soft bottoms in the coastal area of Calabria contributes to a higher trawl accessibility than in the Salento counterpart (Russo et al., 2017). Consequently, the SH shark and rays group (FG 8) showed higher EE value in the CAL than in the SAL model. The high EE value of SL Squids benthopelagic feeders in Salento is due to the high abundance of odontocetes, which are the main predators of squids and cephalopods (Milani et al., 2018; Würtz & Marrale, 1993). Myctophids, Bluntsnout grenadier, SL Bathypelagic fishes piscivorous, and SHB Fishes zooplanktivorous showed higher EE values in the CAL than the SAL food web. Despite their intermediate trophic position in both NWIS food webs, their predation mortality rate was higher in Calabria than in Salento, probably due to their higher vulnerability to consumers linked to the wider occurrence of meso‐bathyal grounds. The higher EE observed for the Loggerhead Turtle in the CAL model could be due to their higher fishing mortality in this area as by‐catch of long lines (Casale, 2011).

Large pelagics, Sea birds, Fin whale, Loggerhead Turtle, and the group of bathyal sharks and rays benthopelagic feeders (FG 9) showed high OI values typical of generalist consumers in both investigated food webs. Small pelagic fishes, SHB_BSquids_BP, and SHB Fishes zooplanktivorus showed low values of OI because they are specialist feeders of zooplankton resources. Similarly, the Anglers and Hake showed a low OI due to high fish predation. The differences in the OI values detected between the CAL and SAL food webs for Odontocetes, SL Octopus and Bobtail Squids benthic feeders, SL Bathypelagic Fishes piscivorous, SL Fishes planktivorous, and SH Sharks and Rays benthic feeders could be explained by dissimilarity in their trophic niches due to variability in the abundance and depth distribution of their preys (Colloca, Carpentieri, Balestri, & Ardizzone, 2010).

The Macrozooplankton and Micro‐Mesozooplankton have been characterized as structural groups due to their high biomass contribution, highlighting their bottom‐up control for both NWIS food webs. This result confirmed a general pattern of trophic flow regulation performed by groups at basal levels due to the oligotrophic conditions of the Mediterranean Sea (Siokou‐Frangou et al., 2010). The role of control of basal levels is also played by the macrobenthic invertebrates, polychaetes, and suprabenthic crustaceans, which show high values of KS in both the investigated food webs, as well as in other Mediterranean models (see Coll & Libralato, 2012). These groups were the main dominant groups due to their high biomasses in both the SAL and CAL models. Therefore, according to Piraino, Fanelli, and Boero (2002), these functional groups could be considered structural components of the investigated food webs.

Clear differences were observed concerning the role of top predators in the Calabrian and Salento models. Anglers were the main keystone predator among bony fishes FGs in the Calabrian food web, similarly to observations in a no‐fishing area of the Central Adriatic Sea (see Coll & Libralato, 2012) and in the cold‐water coral province in the Northern Ionian Sea, where fishing exploitation is limited to avoid damage to gear (Vassallo et al., 2017). In addition, the velvet‐belly *Etmopterus spinax* and kitefin shark *Dalatias licha* were the main species in the FG Slope Sharks benthopelagic feeders identified for its keystone predator role in the Calabria food web, confirming reports for both sharks in the canyon system food‐web model of the Catalan Sea (Tecchio et al., 2013). Both results seem to confirm that top predators species in the demersal and benthopelagic assemblages of NWIS could show their keystone traits and play a top‐down control role only when the food web is scarcely impacted by fishing and/or is characterized by ecological refuges reducing their vulnerability (Fernandez‐Arcaya et al., 2017). Similar observations have previously been reported in the food‐web models provided for the Mediterranean Sea in the North and Central Adriatic Sea, and the Gulf of Gabes (Coll et al., 2007; Hattab et al., 2013), where the fishing exploitation impacts on the key predators determined a clear anthropogenic modification of food webs with consequent impacts on marine ecosystem structure, and functioning (Baum & Worm, 2009; Pauly, Christensen, Dalsgaard, Froese, & Torres, 1998). Moreover, Slope Squids benthopelagic feeders seem to play a more important key role in Calabria than Salento, likely due to the higher biomass in the former food web, with a great trophic impact on their preys (Coll, Navarro, Olson, & Christensen, 2013).

Odontocetes were found to be the main keystone predators in Salento as also observed in other Mediterranean areas (Banaru et al., 2013; Coll et al., 2007). Moreover, an important key role in the Salento food web was also identified for the benthopelagic squids and cephalopods in shelf and shelf break zones as reported in Coll et al. (2013).

The analysis of KSi values and overall relative impacts indicated a bunch of functional groups assuming a wasp‐waist control impacting on the basal and apical groups of the food web (Cury et al.., 2000). The Small pelagics and Benthopelagic Shrimps regulated both food webs through their trophic positions and migrations influencing the availability of energy for many consumers in the shallower and epibathyal zones, respectively. The SHB Fishes benthopelagic crustaceans feeders seems to drive the energy flow from shallower depths toward the bathyal zones in the Calabrian food web. A similar role was detected for the Medium pelagic fishes at shallower depths in the Salento food web.

A complex system of energy and biomass exchanges characterized the investigated food web structures, as detected in other Mediterranean areas, such as the Central Adriatic Sea, the Aegean Sea, the Gulf of Lions and the Strait of Sicily (Agnetta et al., 2019; Banaru et al., 2013; Coll et al., 2007; Tsagarakis et al., 2010), indicating the occurrence of a benthic‐pelagic coupling. In general, benthic‐pelagic coupling is mainly regulated by a direct link between plankton and benthic invertebrates, with many aquatic organisms that contribute to the energy transfer processes between several compartments (sensu Griffiths et al., 2017). In particular, the role of demersal and benthopelagic species in the process varied depending on their life‐history traits and their ontogenetic shifts for feeding and reproductive purposes.

In the NWIS, both biomass and trophic flows were mainly aggregated in the pelagic compartment which was the dominant subsystem in the investigated food webs for consumption, similarly to the results observed in North‐Western Mediterranean area, in the Gulf of Gabes and in the Gulf of Cadiz (Corrales et al., 2015; Hattab et al., 2013; Torres, Coll, Heymans, Christensen, & Sobrino, 2013). This dominance is supported by the presence of a planktonic community and different functional groups of Cetaceans, Small and Large pelagics and Seabirds greatly exploiting the food web productivity due to their high consumption rates. Together to the planktonic and pelagic communities, the regulation of flows between benthic‐pelagic coupling in the NWIS seems to occur through the Benthopelagic Shrimps and Slope Bathypelagic fishes piscivorous due to their wasp‐waist control role in both the investigated food webs. In particular, the vertical movements of species seem to occur from slope to shelf break with a higher intensity in Calabria than in Salento. The geomorphological and hydrographical features could affect the biomass and energy exchanges in this coupling, particularly in driving the differences in the percentage of consumption flows detected between the CAL and SAL food webs. The higher flows of consumption of the benthic compartment observed in the Calabrian food web could be influenced by a widespread presence of deep submarine canyons along the continental edge, which increases the productivity of benthic systems (Garcia et al., 2008). Differently, the flows of consumption in the Salento food web seem to be mainly driven by the planktonic productivity supporting the pelagic, benthopelagic and demersal compartments. This condition could be favored by the large extension of the shelf break zone, where marine pelagic predators, such as cetaceans and seabirds, find foraging habitats showing high densities of occurrence (Carlucci, Ricci, Cipriano, & Fanizza, 2017; Yen, Sydeman, & Hyrenbach, 2004). Moreover, the exclusive presence of cold‐water coral community in the Salento area seem to be linked to this high planktonic productivity sustained by deep‐water masses that flow from the southern Adriatic to northern Ionian (2016Calculli, Capezzuto, Carlucci, Carluccio, Maiorano et al., 2016).

The food‐web models realized for the NWIS represent ideal platforms for the development of analysis with dynamic simulations (Christensen & Walters, 2004). Such tools have shown their potential for exploring the ecological questions linked to food web structure and their usefulness in evaluating alternative management scenarios in an ecosystem context (Link, 2002). Nevertheless, the comparative analysis of two food webs by means of functional groups and their functional traits allowed the general pattern of ecosystem structure and functioning in NWIS to be identified, making it an interesting approach to investigate the marine ecosystem.

## AUTHOR CONTRIBUTIONS

Ricci P., Libralato S., and Carlucci R. conceived the ideas and designed methodology; Ricci P., Carlucci R., Maiorano P., Capezzuto F., Sion L., D'Onghia G., and Tursi A. collected data during surveys. provide contributions in collecting data from literature and critically revised it; Ricci P., Libralato S., Carlucci R., Solidoro C., analyzed input and output data; Ricci P., Libralato S., and Carlucci R. led the writing of the manuscript. All authors contributed critically to the drafts and gave final approval for publication.

## Data Availability

Data supporting this manuscript are presented in the main text and in appendices provided.
