# Supplementary material for: Ecosystem functioning of two marine food webs in the North‐Western Ionian Sea (Central Mediterranean Sea)
Source: Ecol Evol. 2019 Sep 3;9(18):10198–212. doi: 10.1002/ece3.5527 (PMC6787816; doi:10.1002/ece3.5527)
Supplement: Supplementary file 1 [file ECE3-9-10198-s001.docx]

Table S1. Input data and data sources of the Salento and Calabria model in North-western Ionian Sea.

| **FG, input parameter** | **Original value** | **Source** | **Taxa/Notes** |
| --- | --- | --- | --- |
| 1. Odontocetes |  |  | *Stenella coeruleoalba, Tursiops truncatus, Delphinus delphis, Grampus griseus, Delphinidae, Physeter macrocephalus.* |
| Bi (t km^-1^ y^-1^) | CAL 0.0170  SAL 0.0335 | OBIS Sea Map (Halpin et al., 2009); Moutopoulos et al., 2013. | Abunance data were transformed by mean s mean individual weight (Piroddi et al., 2010) |
| P/B (year^-1^) | CAL 0.064  SAL 0.064 | Mackinson et al., 2000; Coll et al., 2007; |  |
| Q/B | CAL 18.760  SAL 18.760 | Trites and Pauly, 1998; Laran et al., 2010 |  |
| Diet |  | Blanco et al., 2001; Evans and Hindell, 2004; Spitz et al., 2006; Bloch et al., 2012; Würtz Marrale, 1993 |  |
| 2. F whale |  |  | *Balenoptera physalus.* |
| Bi (t km^-1^ y^-1^) | CAL 0.0064  SAL 0.0123 | OBIS Sea Map (Halpin et al., 2009) | Abunance data were transformed by mean s mean individual weight (Piroddi et al., 2010) |
| P/B | CAL 0.040  SAL 0.040 | Coll et al., 2008 |  |
| Q/B | CAL 4.110 | Trites and Pauly, 1998; Laran et al., 2010 |  |
| Diet | SAL 4.110 | Pauly et al., 1998 |  |
| 3. Log turtle |  |  | *Caretta caretta.* |
| Bi (t km^-1^ y^-1^) | CAL 0.038  SAL 0.074 | OBIS Sea Map (Halpin et al., 2009; Moutopoulos et al., 2013 |  |
| P/B | CAL 0.270  SAL 0.270 | Casale et al., 2007 |  |
| Q/B | CAL 3.500  SAL 3.500 |  |  |
| Diet |  | Tomas et al., 2001; Casale et al. 2008; |  |
| 4. Seabirds |  |  | *Larus michahellis, Larus ridibundus, Phalacrocorax carbo, Puffinus puffinus.* |
| Bi (t km^-1^ y^-1^) | CAL 0.0008  SAL 0.0012 | Baccetti et al. (2002);  Zenatello, Baccetti & Borghesi (2014) |  |
| P/B | CAL 0.140  SAL 0.140 | Ristow et al., 1990. |  |
| Q/B | CAL 60.830  SAL 60.830 | Nagy, 1878 |  |
| Diet |  | Fasola et al., 1989 |  |
| 5. L pelagics |  |  | *Alopias vulpinus, Prionace glauca, Thunnus alalunga, Xiphias gladius, Seriola dumerili.* Pelagic |
| Bi (t km^-1^ y^-1^) | CAL 0.0247  SAL 0.0575 | Piroddi et al., 2015, Moutopoulos et al., 2013; Agnetta et al., 2019 |  |
| P/B | CAL 0.875  SAL0.875 | Moutopoulos et al., 2013 | Weighted on average local biomass of the species group |
| Q/B | CAL 3.270  SAL 3.270 | Moutopoulos et al., 2013, Froese and Pauly, 2008 | www.fishbase.org, estimated on average local biomass of the species group using empircal equation |
| Diet |  | Bello, 1991; Karakulak et al., 2009; Battaglia et al., 2013. |  |
| 6. SL_SharkRays_bent |  |  | *Cetrophorus granulosus, Dipturus oxyrinchus, Leucoraja circularis, Leucoraja fullonica, Torpedo nobiliana.* Demersal |
| Bi (t km^-1^ y^-1^) | CAL 0.0110  SAL 0.0121 | Trawl Survey (MEDITS) | Average biomass 1995-1997 |
| P/B | CAL 0.580  SAL 0.593 | Pauly, 1980, Lopez, 2013 | Z=F+M Empirical equation |
| Q/B | CAL 2.000  SAL 2.000 | Froese and Pauly, 2008, Lopez, 2013 | www.fishbase.org, estimated on average local biomass of the species group using empircal equation |
| Diet |  | Macpherson, 1981; Megalofonu and Chatzispyrou, 2006; Yign et al., 2010. |  |
| 7. SH-SHB_SharkRays_BP |  |  | *Gymnura altavela, Raja miraletus, Scyliorhinus canicola, Scyliorhinus stellaris* |
| Bi (t km^-1^ y^-1^) | CAL 0.0058  SAL 0.0016 | Trawl Survey (MEDITS) | Average biomass 1995-1997 |
| P/B (year^-1^) | CAL 0.665  SAL 0.600 | Pauly, 1980 | Z=F+M Empirical equation |
| Q/B (year^-1^) | CAL 3.150  SAL 3.400 | Pauly et al., 1990, Froese and Pauly, 2008 | www.fishbase.org, estimated on average local biomass of the species group using empircal equation |
| Diet |  | Vannucci et al., 2006. |  |
| 8. SH_SharkRays_bent |  |  | *Dasyatis centroura, Dasyatis pastinaca, Mustelus mustelus, Pteromylaeus bovinus, Raja asterias, Torpedo marmorata, Torpedo torpedo* |
| Bi (t km^-1^ y^-1^) | CAL 0.1427  SAL 0.0404 | Trawl Survey (MEDITS) | Average biomass 1995-1997 |
| P/B | CAL 0.810  SAL 0.785 | Pauly, 1980 | Z=F+M Empirical equation |
| Q/B | CAL 3.600  SAL 3.600 | Pauly et al., 1990, Froese and Pauly, 2008 | www.fishbase.org, estimated on average local biomass of the species group using empircal equation |
| Diet |  | Morte et al., 1997; Cortes, 1999; Ismen, 2003; Romanelli et al., 2006; Yeldan et al., 2009. |  |
| 9. SL_Sharks_BP |  |  | *Chimaera monstrosa, Dalatias licha, Etmopterus spinax, Squalus blainvillei* |
| Bi (t km^-1^ y^-1^) | CAL 0.0569  SAL 0.0284 | Trawl Survey (MEDITS) | Average biomass 1995-1997 |
| P/B | CAL 0.520  SAL 0.675 | Pauly, 1980, STECF, 2011. | Z=F+M Empirical equation |
| Q/B | CAL 2.177  SAL 8.026 | Pauly et al., 1990, Froese and Pauly, 2008 Madurell and Cartes, 2005 | www.fishbase.org, estimated on average local biomass of the species group using empircal equation |
| Diet |  | Macpherson, 1981; Smale, 1996; Belluscio et al., 2000. |  |
| 10. B catshark |  |  | *Galeus melastomus* |
| Bi (t km^-1^ y^-1^) | CAL 0.0199  SAL 0.0122 | Trawl Survey (MEDITS) | Average biomass 1995-1997 |
| P/B | CAL 0.650  SAL 0.650 | Pauly, 1980, STECF, 2011 | Z=F+M Empirical equation |
| Q/B | CAL 5.431  SAL 5.431 | Madurell and Cartes, 2005 |  |
| Diet |  | Carrassón, et al., 1992 |  |
| 11. SL_DemF_opp |  |  | *Conger conger, Molva dypterigia, Polyprion americanus* |
| Bi (t km^-1^ y^-1^) | CAL 0.0332  SAL 0.0321 | Trawl Survey (MEDITS) | Average biomass 1995-1997 |
| P/B | CAL 0.613  SAL 0.648 | Merz and Myers, 1998, Ainsworth et al., 2002, Diaz Lopez et al., 2008 | estimated on average local biomass of the species group |
| Q/B | CAL 2.107  SAL 2.074 | Pauly et al., 1990, Froese and Pauly, 2008 | www.fishbase.org, estimated on average local biomass of the species group using empircal equation |
| Diet |  | Macpherson, 1981; Bergstad, 1991; Olaso et al., 1995; Morato et al., 1999; O'Sullivan et al., 2004; |  |
| 12. SHB-SL_DemF_gen feed |  |  | *Gaidropsaurus biscayensis, Pagellus bogaraveo, Peristedion cataphractum, Phycis phycis, Trigla lyra* |
| Bi (t km^-1^ y^-1^) | CAL 0.0550  SAL 0.0211 | Trawl Survey (MEDITS) | Average biomass 1995-1997 |
| P/B | CAL 1.157  SAL 1.035 | Pauly, 1980, Colloca et al., 1997, Merz and Myers, 1998 | Z=F+M Empirical equation, www.fishbase.org |
| Q/B | CAL 4.307  SAL 4.307 | Pauly et al., 1990, Froese and Pauly, 2008, Carpentieri et al., 2007 | www.fishbase.org, estimated on average local biomass of the species group using empircal equation |
| Diet |  | Macpherson, 1981, Morato et al., 1999, 2001, |  |
| 13. SH-SHB_DemF_gen feed |  |  | *Ariosoma balearicum, Aspitrigla cuculus, Carapacus acus, Dactylopterus volitans, Dentex dentex, Dentex gibbosus, Echelus myrus, Epinephelus aeneus, Eutrigla gurnardus, Lepidorhombus whiffiagonis, Ophidion barbatum, Pagellus erythrinus, Scorpaena porcus, Serranus cabrilla, Serranus hepatus, Sphoeroides pachygaster, Synodus saurus, Trachinus draco, Trigloporus lastoiviza, risopterus minutus, Uranoscopus scaber, Zeus faber* |
| Bi (t km^-1^ y^-1^) | CAL 0.2896  SAL 0.1318 | Trawl Survey (MEDITS) | Average biomass 1995-1997 |
| P/B | CAL 0.711  SAL 0.841 | Pauly, 1980, Colloca et al., 1997, Merz and Myers, 1998, Arreguìn-Sanchèz et al., 2002 | Z=F+M Empirical equation, www.fishbase.org |
| Q/B | CAL 3.372  SAL 4.573 | Pauly et al., 1990, Stergiou, 1991, Froese and Pauly, 2008, Ainsworth et al., 2002, Carpentieri et al., 2007. | www.fishbase.org, estimated on average local biomass of the species group using empircal equation |
| Diet |  | Sanz, 1985, Moreno-Amich, 1992, 1994, Labropoulou et al., 1997, Morte et al., 1999, Terrats et al., 1999, Soares et al., 2003, Samir, 2008, Başçınar et al., 2009, Fanelli et al., 2011, |  |
| 14. SH-SHB_DemF_pisc |  |  | *Micromesistius poutassou, Scorpaena elongata, Scorpaena notata, Scorpaena scrofa* |
| Bi | CAL 0.2736  SAL 0.1268 | Trawl Survey (MEDITS) | Average biomass 1995-1997 |
| P/B | CAL 1.656  SAL 1.760 | Pauly, 1980, Merz and Myers, 1998 | Z=F+M Empirical equation, www.fishbase.org |
| Q/B | CAL 7.300  SAL 6.959 | Pauly et al., 1990, Froese and Pauly, 2008 | www.fishbase.org, estimated on average local biomass of the species group using empircal equation |
| Diet |  | Kyrtatos, 1982, Harmelin Vivien et al., 1989, Bradai et al., 1990, Olaso et al., 1995, Relini, 2002, |  |
| 15. SL_BathypelF_pisc |  |  | *Chauliodus sloani, Evermanella balbo, Gonostoma denudatum, Lampanyctus crocodilus, Nettastoma melanurum, Paralepis speciosa, Stomias boa, Sudis hyalina* |
| Bi | CAL 0.1283  SAL 0.1239 |  | Trawl Survey (MEDITS) Average biomass 1995-1997 |
| P/B | CAL 3.347  SAL 2.619 | Arreguìn-Sànchez et al., 2002, Stanford et al., 2002; Ainsworth et al., 2002, Guènette et al., 2002, Heymans, 2005, Rosas-Luis et al., 2008 | www.fishbase.org |
| Q/B | CAL 10.209  SAL 7.604 | Childress et al., 1980, Pauly et al., 1990, Froese and Pauly, 2008 | www.fishbase.org; estimated on average local biomass of the species group using empircal equation |
| Diet |  | Stefanescu et al., 1992, Hopkins et al., 1996 |  |
| 16. SL_DemF_shrimps feed |  |  | *Centrolophus niger, Chlopsis bicolor, Cubiceps gracilis, Epigonus telescopus, Gadella maraldi, Gnatophis mystax, Helycolenus dactylopterus, Lepidorhombus boscii, Ophisurus serpens, Phycis blennoides.* |
| Bi | CAL 0.0972  SAL 0.0751 |  | Trawl Survey (MEDITS) Average biomass 1995-1997 |
| P/B | CAL 1.205  SAL 1.179 | Beverton et al., 1957, Pauly, 1980, Merz and Myers, 1998 Guènette et al., 2002, Heymans, 2005, Griffiths et al., 2010 | Z=F+M Empirical equation, www.fishbase.org |
| Q/B | CAL 4.524  SAL 4.241 | Pauly et al., 1990, Froese and Pauly, 2008, Carpentieri et al., 2007 | www.fishbase.org, estimated on average local biomass of the species group using empircal equation |
| Diet |  | Macpherson, 1981, Morte et al., 2002, Vassilopoulou, 2006, Carpentieri et al., 2007, Consoli et al., 2010, |  |
| 17. SL_F_BP crust feed |  |  | *Trachyrhynchus scabrus, Argyropelecus hemigymnus, Nemichthys scolopacues, Microichthys coccoi, Epigonus constancie, Epigonus denticulatus, Gonichthys coccoi* |
| Bi | CAL 0.3460  SAL 0.3460 | Trawl Survey (MEDITS) | Average biomass 1995-1997 |
| P/B | CAL 1.296  SAL 1.124 | Pauly, 1980, Silvestre et al., 1993, Vega-Cendejas et al., 1993, Merz and Myers, 1998, Guènette et al., 2002, Stanford et al., 2002, Anastasopoulou et al., 2006 | Z=F+M Empirical equation, www.fishbase.org |
| Q/B | CAL 5.739  SAL 5.019 | Pauly et al., 1990, Carpentieri et al., 2007, Froese and Pauly, 2008 | www.fishbase.org; estimated on average local biomass of the species group using empircal equation |
| Diet |  | Macpherson, 1981, Hopkins et al., 1985 |  |
| 18. SHB_F_BP crust feed |  |  | *Argentina sphyrena, Arnoglossus rueppelli, Aulopus filamentosus, Clorophtalmus agasizii, Dalophis imperbis, Echiodon dentatus, Gadiculus argenteus, Gobius niger, Ichthyococcus ovatus, Lepidopus caudatus, Leusuerigobius suerii, Maurolicus muelleri, Ophicthus rufus, Symphurus ligualtus, Symphurus nigrescens, Vinciuerria attenuata* |
| Bi | CAL 0.4885  SAL 0.7276 | Trawl Survey (MEDITS) | Average biomass 1995-1997 |
| P/B | CAL 1.780  SAL 0.746 | Beverton and Holt, 1957, Pauly, 1980, Guénette et al., 2001, Brando et al., 2004 | Z=F+M Empirical equation, www.fishbase.org |
| Q/B | CAL 8.155  SAL 3.078 | Pauly et al., 1990, Carpentieri et al., 2007, Froese and Pauly, 2008 | www.fishbase.org, estimated on average local biomass of the species group using empircal equation |
| Diet |  | Macpherson, 1979, 1981, Bell et al., 1983, Gorelova et al., 1990, Meyer et al., 1991, Casadevall et al., 1994, Longo et al., 2005, Anastasopoulou et a., 2008 |  |
| 19. SH_DemF_bent crust feed |  |  | *Aphia minuta, Arnoglossus laterna, Arnoglossus thori, Boops boops, Callionymus fasciatus, Callionymus maculatus, Centracanthus cirrus, Cepola macrophthalma, Chelydonichthys lucerna, Chelydonichthys obscurus, Deltentosteus quadrimaculatus, Diplodus annularis, Gobius geniporus, Hippocampus hippocampus, Lepidotrigla cavillone, Spiacara maena (flexuosa), Spicara smaris, Symphodus cinereus, Synchiropus phaeton, Syngnathus acus* |
| Bi | CAL 0.4451  SAL 0.2833 | Trawl Survey (MEDITS) | Average biomass 1995-1997 |
| P/B | CAL 1.221  SAL 0.857 | Pauly, 1980, Colloca et al., 1997, Brando et al. 2004, | Z=F+M Empirical equation, www.fishbase.org |
| Q/B | CAL 6.587  SAL 5.876 | Pauly et al., 1990, Froese and Pauly, 2008 | www.fishbase.org, estimated on average local biomass of the species group using empircal equation |
| Diet |  | Juki, 1972, Khoury, 1984, Rosecchi, 1987, Stergiou, 1993, Labropoulou et al., 1998, Terrats et al., 1999, Cabral et al., 2002, Fanelli et al., 2009, Tuncay et a., 2010, Stagioni, 2012 |  |
| 20. SH_DemF_bent inv feed |  |  | *Blennius ocellaris, Bothus podas, Buglossidium luteum, Callanthias ruber, Citharus linguatula, Dicologlossa cuneata, Diplodus vulgaris, Lithognathus mormyrus, Leusuerigobius friesii, Microchirus ocellatus, Monochirus hispidus, Mullus surmuletus, Pagellus acarne, Pagrus pagrus, Sparus aurata, Xyrichtys novacula* |
| Bi | CAL 0.4451  SAL 0.1402 | Trawl Survey (MEDITS) | Average biomass 1995-1997 |
| P/B | CAL 0.825  SAL 0.930 | Pauly, 1980, Brando et al. 2004, STECF, 2011 | Z=F+M Empirical equation, www.fishbase.org |
| Q/B | CAL 4.898  SAL 5.186 | Pauly et al., 1990, Carpentieri et al., 2006, Froese and Pauly, 2008 | www.fishbase.org; estimated on average local biomass of the species group using empircal equation |
| Diet |  | Cardinale et al., 1997, Sala et al., 1997, Šantić, 2010, Fanelli et al., 2011, |  |
| 21. SL_F_planktivorous |  |  | *Lepidion lepidion, Mora moro* |
| Bi | CAL 0.0168  SAL 0.0333 | Trawl Survey (MEDITS) | Average biomass 1995-1997 |
| P/B | CAL 0.840  SAL 0.840 | Pauly, 1980 | Z=F+M Empirical equation, www.fishbase.org |
| Q/B | CAL 3.000  SAL 3.000 | Pauly et al., 1990, Froese and Pauly, 2008 | www.fishbase.org, estimated on average local biomass of the species group using empircal equation |
| Diet |  | Carrassón et al., 1997 |  |
| 22. SHB_F_planktivorous |  |  | *Benthocomets robustus, Capros aper, Glossanodon leioglossus, Gymnammodytes cicerellus, Macroramphosus scolopax* |
| Bi | CAL 0.5778  SAL 0.2175 | Trawl Survey (MEDITS) | Average biomass 1995-1997 |
| P/B | CAL 1.347  SAL 1.009 | Beverton and Holt, 1957, Guénette et al., 2001, Ainsworth et al., 2002 | www.fishbase.org; estimated on average local biomass of the species group using empircal equation |
| Q/B | CAL 6.951  SAL 6.987 | Pauly et al., 1990, Froese and Pauly, 2000, Carpentieri et al., 2006 | www.fishbase.org; estimated on average local biomass of the species group using empircal equation |
| Diet |  | Macpherson, 1979, Longo et al., 2005 |  |
| 23. S pelagics |  |  | *Sprattus sprattus, Engraulis encransicolus, Sardinella aurita, Sardina pilchardus* |
| Bi | CAL 0.8311  SAL 0.8213 | Trawl Survey (MEDITS) | Maximum values of biomass 1995-1996 |
| P/B | CAL 1.044  SAL 1.429 | Pauly, 1980, STECF, 2011. | Z=F+M Empirical equation, www.fishbase.org |
| Q/B | CAL 8.644  SAL 8.459 | Pauly et al., 1990, Froese and Pauly, 2008, Tudela et al., 1995, Plounevez et al., 2000, | www.fishbase.org; estimated on average local biomass of the species group using empircal equation |
| Diet |  | Sirotenko et al., 1979, Sever et al., 2005, Bacha et al., 2010, |  |
| 24. M pelagics |  |  | *Scomber japonicus, Trachurus picturatus, Trachurus trachurus, Scomber scombrus, Trachurus mediterraneus, Sphyrena sphyrena* |
| Bi | CAL 0.4953  SAL 0.2881 | Trawl Survey (MEDITS) | Average biomass 1995-1997 |
| P/B | CAL 1.300  SAL 1.229 | Pauly, 1980 | Z=F+M Empirical equation, www.fishbase.org |
| Q/B | CAL 3.507  SAL 3.999 | Pauly et al., 1990, Froese and Pauly, 2008 | www.fishbase.org; estimated on average local biomass of the species group using empircal equation |
| Diet |  | Cabral et al., 2002, Šantić et al., 2003, 2005, Jardas et al., 2004, Campo et al., 2006, Sever et al., 2006 |  |
| 25. Macrourids |  |  | *Caelorhyncus caelorhynchus, Hymenocephalus italicus, Notacanthus bonapartei, Hoplostetus mediterraneus, Symbolophorus veranyi, Bathypterois dubius* |
| Bi | CAL 0.7654  SAL 0.2582 | Trawl Survey (MEDITS) | Average biomass 1995-1996 |
| P/B | CAL 0.552  SAL 2.2728 | Pauly, 1980, Tsarin, 1994, Arreguín-Sánchez et al., 2002, Stanford et al., 2002, Heymans, 2005, Rosas-Luis et al., 2009 | Z=F+M Empirical equation, www.fishbase.org |
| Q/B | CAL 3.797  SAL 5.935 | Pauly et al., 1990, Pakhomov et al., 1996, Madurell and Cartes., 2005, Froese and Pauly, 2008 | www.fishbase.org; estimated on average local biomass of the species group using empircal equation |
| Diet |  | Macpherson, 1979, Podrazhanskaya, 1993, Carrassón et al., 2002, Madurell and Cartes, 2005, Tuncay et al., 2008, |  |
| 26. Myctophids |  |  | *Bethosema glaciale, Ceratoscopelus maderensis, Electrona rissoi, Myctophum punctatum, Hygophum hygomii, Diaphus rafinesqui, Diaphus metopoclampus, Diaphus holti, Lobianchia dofleini, Hygophum benoiti, Notoscopelus bolini, Notoscopelus elongatus, Myctophidae* |
| Bi | CAL 0.3513  SAL 0.680 | Trawl Survey (MEDITS) | Average biomass 1995-1997 |
| P/B | CAL 4.045  SAL 4.700 | Tsarin, 1994, Arreguín-Sánchez et al., 2002, Stanford et al., 2002, Heymans, 2005, Rosas-Luis et al., 2009 |  |
| Q/B | CAL 10.088  SAL 10.290 | Froese and Pauly, 2008, Pakhomov et al., 1996, | www.fishbase.org; estimated on average local biomass of the species group using empircal equation |
| Diet |  | Podrazhanskaya, 1993, Pakhomov et al., 1996, |  |
| 27. R mullet |  |  | *Mullus barbatus* |
| Bi | CAL 0.0561  SAL 0.0223 | Trawl Survey (MEDITS) | Average biomass 1995-1997 |
| P/B | SAL 1.190  CAL 1.190 | Pauly, 1980, STECF, 2011, Agnetta et al., 2019 | Z=F+M Empirical equation, www.fishbase.org |
| Q/B | CAL 4.325  SAL 4.325 | Carpentieri et al., 2007, Maioarano et al., 2010 |  |
| Diet |  | Labrapoulou et al., 1997 |  |
| 28. Hake |  |  | *Merluccius merluccius* |
| Bi | CAL 0.0413  SAL 0.500 | Trawl Survey (MEDITS) | Average biomass 1995-1997 |
| P/B | CAL 1.792  SAL 2.570 | Pauly, 1980, STECF, 2011, Maioarano et al., 2010 | Z=F+M Empirical equation, www.fishbase.org |
| Q/B | SAL 8.614  CAL 6.640 | Madurell and Cartes, 2005; Agnetta e al., 2019 |  |
| Diet |  | Bozzano et al., 1997; Stagioni et al., 2011 |  |
| 29. Anglers |  |  | *Lophius budegassa, L. piscatorius* |
| Bi (t km^-2^ y^-1^) | CAL 0.0078  SAL 0.0144 | Trawl Survey (MEDITS) | Average biomass 1995-1997 |
| P/B | CAL 0.920  SAL 0.920 | Corrales et al., 2015, Maioarano et al., 2010, Carlucci et al., 2009. |  |
| Q/B | CAL 4.03  SAL 4.03 | Corrales et al., 2015 |  |
| Diet |  | Velasco et al., 1996, Stagioni, 2013 |  |
| 30. Blunt grenad |  |  | *Nezumia sclerorhyncus* |
| Bi (t km^-2^ y^-1^) | CAL 0.0441  SAL 0.0537 | Trawl Survey (MEDITS) | Average biomass 1995-1997 |
| P/B | CAL 1.110  SAL 1.110 | Beverton and Holt, 1957 | www.fishbase.org; estimated on average local biomass of the species group using empircal equation |
| Q/B | CAL 4.500  SAL 4.500 | Pauly et al., 1990, Froese and Pauly, 2008 | www.fishbase.org; estimated on average local biomass of the species group using empircal equation |
| Diet |  | Macpherson, 1979 |  |
| 31. SL_Squids_BP |  |  | *Onychoteuthis banksii, Ancistroteuthis lichtensteinii, Histioteuthis bonnellii, Histioteuthis reversa, Todarodes sagittatus, Ancistroteuthis lesuerii, Pyroteuthis margarifera, Brachioteuthis riisei, Chiroteuthis veranii* |
| Bi | CAL 0.0482  SAL 0.0217 | Trawl Survey (MEDITS) | Average biomass 1995-1997 |
| P/B | CAL 2.176  SAL 2.222 | Wells & Clarke, 1996, Brey, 2001 | Empirical equation |
| Q/B | CAL 12.024  SAL 10.474 | Cammen, 1980 | Empirical equation |
| Diet |  | Quetglas et al., 1999, 2010 |  |
| 32. SHB_Squids_BP |  |  | *Abralia verany, Todaropsis eblanae, Loligo forbesii, Illex coindetii, Alloteuthis media, Loligo vulgaris, Allo teuthis subdulata* |
| Bi | CAL 0.0426  SAL 0.0485 | Trawl Survey (MEDITS) | Average biomass 1995-1997 |
| P/B | CAL 4.511  SAL 4.452 | Wells & Clarke, 1996, Brey, 2001 | Empirical equation |
| Q/B | CAL 12.935  SAL 13.991 | Cammen, 1980 | Empirical equation |
| Diet |  | Guerra et al., 1994, Castro et al., 1995, Coelho et al., 1996, Rasero et al., 1996 |  |
| 33. SH_Ceph_BP |  |  | *Scaeurgus unicirrhus, Sepia orbignyana, Eledone moschata, Eledone cirrhosa, Sepia elegans, Sepiola rondoletii, Ocotopus vulgaris, Sepiola robusta, Sepiola intermedia, Sepia officinalis, Octopus macorpus* |
| Bi (t km^-2^ y^-1^) | CAL 0.1041  SAL 0.1147 | Trawl Survey (MEDITS) | Average biomass 1995-1997 |
| P/B | CAL 5.770  SAL 5.334 | Wells & Clarke, 1996, Brey, 2001 | Empirical equation |
| Q/B | CAL 22.481  SAL 19.320 | Cammen, 1980 | Empirical equation |
| Diet |  | Castro et al., 1990, Rosa et al., 2004, Sifner et al., 2009, Quetglas et al., 1998 |  |
| 34. SL_Octopus_bent |  |  | *Octopoteuthis sicula, Neorossia caroli, Heteroteuthis dispar, Octopus salutii, Pteroctopus tetracirrhus* |
| Bi (t km^-2^ y^-1^) | CAL 0.0312  SAL 0.0431 | Trawl Survey (MEDITS) | Average biomass 1995-1997 |
| P/B | CAL 3.661  SAL 3.636 | Brey, 2001 | Empirical equation |
| Q/B | CAL 9.926  SAL 9.625 | Cammen, 1980 | Empirical equation |
| Diet |  | Quetglas et al., 2005, 2009 |  |
| 35. SHB_BSquids_BP |  |  | *Rondoletia minor, Sepietta oweniana, Rossia macrosoma, Sepiola ligulata* |
| Bi (t km^-1^ y^-1)^ | CAL 0.0338  SAL 0.0597 | Trawl Survey (MEDITS) | Average biomass 1995-1997 |
| P/B | CAL 5.372  SAL 4.763 | Brey, 2001 | Empirical equation |
| Q/B | CAL 18.235  SAL 16.773 | Cammen, 1980 | Empirical equation |
| Diet |  | Bergstrom, 1985 |  |
| 36. Shrimps BP |  |  | *Acanthephyra eximia, Acanthephyra pelagica, Chlorotocus crassicornis, Deosergestes arachnipodus, Ligur ensifer, Pasipahea multidentata, Pasiphaea sivado, Philocheras echinulatus, Plesionka edwarsii, Plesionika gigliolii, Plesionka spp., Polycheles typhlops, Processa canaliculata, Processidae, Rissoides desmaresti, Rissoides pallidus, Sergestidae, Sergia robusta* |
| Bi (t km^-1^ y^-1)^ | CAL 0.3928  SAL 0.6192 | Trawl Survey (MEDITS) | Average biomass 1995-1997 |
| P/B | CAL 3.221  SAL 3.750 | Brey, 2001 | Empirical equation |
| Q/B | CAL 11.760  SAL 11.326 | Cammen, 1980, Maynou and Cartes, 1998 | Empirical equation |
| Diet |  | Cartes, 1992, 1993a,b, Fanelli et al., 2004 |  |
| 37. SL_Decap_Scav |  |  | *Anamathia rissoana, Geryon longipes, Parthenope macrochelos, Plesionka acanthonotus, Bathynectes maravigna, Paromola cuvieri, Monadaeus couchii, Munida tenuimana, Munida rugosa, Munida intermedia, Munida rutllanti (iris), Nephrops norvegicus, Aegeon lacazei, Solenocera membranacea, Pontophilus spinosus, Plesionka heterocarpus, plesionka antigai, Munida spp.,* |
| Bi (t km^-1^ y^-1)^ | CAL 0.2025  SAL 0.2735 | Trawl Survey (MEDITS) | Average biomass 1995-1997 |
| P/B | CAL 2.664  SAL 2.682 | Brey, 2001, Maioarano et al., 2010. | Empirical equation and Z (Total Mortality) for *Nephrops norvegicus* |
| Q/B | CAL 9.920  SAL 8.065 | Cammen, 1980, Maynou and Cartes, 1998 | Empirical equation |
| Diet |  | Cartes, 1993c,d, 1995, Cristo, 1998, Fanelli et al., 2004 |  |
| 38. SL_Crabs |  |  | *Goneplax rhomboides, Pagurus alatus, Macropipus tuberculatus* |
| Bi (t km^-1^ y^-1^) | CAL 0.1139  SAL 0.1728 | Trawl Survey (MEDITS) | Average biomass 1995-1997 |
| P/B | CAL 2.322  SAL 2.333 | Brey, 2001 | Empirical equation |
| Q/B | CAL 10.287  SAL 10.311 | Cammen, 1980, Maynou and Cartes, 1998 | Empirical equation |
| Diet |  | Abelló, 1989 |  |
| 39. SHB_Crabs |  |  | *Macropodia longipes, Maja crispata, Maja squinado, Latreilla elegans, dardanus arrosor, Homola barbata, Plesionka narval, Alpheus glaber* |
| Bi (t km^-1^ y^-^) | CAL 0.1919  SAL 0.2933 | Trawl Survey (MEDITS) | Average biomass 1995-1997 |
| P/B | CAL 2.690  SAL 2.327 | Brey, 2001 | Empirical equation |
| Q/B | CAL 11.780  SAL 10.335 | Cammen, 1980, Maynou and Cartes, 1998 | Empirical equation |
| Diet |  | Bernardez et al., 2000 |  |
| 40. SH_Crabs |  |  | *Medorippe longipes, Liocarcinus depurator, Calappa granulata, Calappa rissoana, Calappa turkeyana,Scyllarides latus, Macropodia rostrata, Atelecyclus rotundatus, Inachus comunissimus, Inachus dorsettensis, Squilla mantis.* |
| Bi (t km^-1^ y^-^) | CAL 0.8680  SAL 0.198 | Trawl Survey (MEDITS) |  |
| P/B | CAL 2.470  SAL 2.459 | Brey, 2001, STECF, 2011, | Empirical equation |
| Q/B | CAL 10.932  SAL 11.798 | Cammen, 1980 | Empirical equation |
| Diet |  | Freire, 1996 | Average biomass 1995-1997 |
| 41. DWR shrimp |  |  | *Parapenaeus longirostris* |
| Bi (t km^-1^ y^-^) | CAL 0.2084  SAL 0.1342 | Trawl Survey (MEDITS) | Average biomass 1995-1997 |
| P/B | CAL 1.413  SAL 1.343 | Brey, 2001, STECF, 2011, Maioarano et al., 2010. | Empirical equation |
| Q/B | CAL 8.867  SAL 11.479 | Cammen, 1980 | Empirical equation |
| Diet |  | Kapiris, 2004 |  |
| 42. RG shrimp |  |  | *Aristaemorpha foliacea* |
| Bi (t km^-1^ y^-^) | CAL 0.0617  SAL 0.0447 | Trawl Survey (MEDITS) | Average biomass 1995-1997 |
| P/B | CAL 1.165  SAL 1.165 | Brey, 2001, STECF, 2011, Maioarano et al., 2010. | Empirical equation |
| Q/B | CAL 7.318  SAL 7.318 | Maynou and Cartes, 1998 | Empirical equation |
| Diet |  | Cartes, 1995 |  |
| 43. BR shrimp |  |  | *Aristaeus antennatus* |
| Bi (t km^-1^ y^-^) | CAL 0.1019  SAL 0.0937 | Trawl Survey (MEDITS) | Average biomass 1995-1997 |
| P/B | CAL 0.726  SAL 0.527 | Brey, 2001, STECF, 2011, Maioarano et al., 2010. |  |
| Q/B | CAL 7.318  SAL 7.318 | Maynou and Cartes, 1998 |  |
| Diet |  | Kapiris et al., 2011 |  |
| 44. G shrimp |  |  | *Plesionika martia* |
| Bi (t km^-1^ y^-^) | CAL 0.1165  SAL 0.1017 | Trawl Survey (MEDITS) | Average biomass 1995-1997 |
| P/B | CAL 2.741  SAL 2.741 | Brey, 2001 |  |
| Q/B | CAL 12.571  SAL 12.571 | Cammen, 1980, Maynou and Cartes, 1998 |  |
| Diet |  | Cartes, 1993a |  |
| 45. Polychaets |  |  | Polychaeta, Nematoda. |
| Bi | - |  | Estimated by model |
| P/B | CAL 5.140  SAL 5.140 |  |  |
| Q/B | CAL 20.708  SAL 20.708 |  |  |
| Diet |  | Fauchald et al., 1979, Hattab et al., 2013 |  |
| 46. Macrobent inv |  |  | Cnidaria, Sipunculida, Mollusca bivalvia, Mollusca gasteropoda, Mollusca scaphopoda, Bryozoa, Echinodermata, Hydrozoa, Ascidiacea, Porifera, Nudibranchs, Hydrozoa, Cirripeda. Benthic-demersal |
| Bi (t km^-1^ y^-^) | - |  | Estimated by model |
| P/B | CAL 5.306  SAL 5.306 |  |  |
| Q/B | CAL 22.536  SAL 22.536 |  |  |
| Diet |  | Lopez, 2013, Hattab et al., 2013, Corrales et al., 2015 |  |
| 47. Gel plank |  |  | Scyphozoa, Siphonophora, Thaliacea. |
| Bi (t km^-1^ y^-^) | - |  | Estimated by model |
| P/B | CAL 13.570  SAL 13.570 |  |  |
| Q/B | CAL 48.800  SAL 48.800 |  |  |
| Diet |  | Tsagarakis et al., 2010, Bănaru et al., 2013 |  |
| 48. Supbent crust |  |  | Cumacea, Ostracoda, Isopoda, Tanaidacea, Amphipoda. |
| Bi (t km^-2^ y^-1^) | - |  | Estimated by model |
| P/B | CAL 7.730  SAL 7.730 |  |  |
| Q/B | CAL 36.510  SAL 36.510 |  |  |
| Diet |  | Bănaru et al., 2013 |  |
| 49. Macrozooplank |  |  | Chetognata, Mysidiacea, Mollusca Pteropoda, Euphasiacea. |
| Bi (t km^-1^ y^-^) | - |  | Estimated by model |
| P/B | CAL 18.000  SAL 18.000 | Lazzari et al., 2012 | Model OPATM-BFM |
| Q/B | CAL 66.000  SAL 66.000 | Lazzari et al., 2012 | Model OPATM-BFM |
| Diet |  | Tsagarakis et al., 2010,  Bănaru et al., 2013 |  |
| 50. Mesozooplank |  |  | Copepoda, Cladocera. |
| Bi (t km^-1^ y^-^) | - |  | Estimated by model |
| P/B | CAL 28.550  SAL 28.550 | Lazzari et al., 2012 | Model OPATM-BFM |
| Q/B | CAL 107.460  SAL 107.460 | Lazzari et al., 2012 | Model OPATM-BFM |
| Diet |  | Tsagarakis et al., 2010, Bănaru et al., 2013 |  |
| 51. Microzooplank |  |  | Foraminifera, Larvae. |
| Bi (t km^-1^ y^-^) | CAL 3.700  SAL 3.700 | Lazzari et al., 2012 | Model OPATM-BFM |
| P/B | CAL 20.870  SAL 20.870 | Lazzari et al., 2012; Agnetta et al., 2019 | Model OPATM-BFM |
| Q/B | CAL 80.730  SAL 80.730 | Lazzari et al., 2012; Agnetta et al., 2019 | Model OPATM-BFM |
| Diet |  | Tsagarakis et al., 2010, Bănaru et al., 2013 |  |
| 52. Bact plank |  |  | Picoplankton. Pelagic |
| Bi (t km^-1^ y^-^) | CAL 6.000  SAL 6.000 | Lazzari et al., 2012 | Model OPATM-BFM |
| P/B | CAL 25.870  SAL 25.870 | Lazzari et al., 2012; Agnetta et al., 2019 | Model OPATM-BFM |
| Q/B | CAL 89.780  SAL 89.780 | Lazzari et al., 2012; Agnetta et al., 2019 | Model OPATM-BFM |
| Diet |  | Bănaru et al., 2013 |  |
| 53. Seagrasses-algae |  |  | *Cymodocea nodosa, Posidonia oceanica,* macrobenthic algae. |
| Bi (t km^-1^ y^-^) | CAL 0.510  SAL 0.590 | ARPA Puglia data, 2009-2011, Buia and Marzocchi, 1995 Banaru et al., 2013 | Posidonia biomass data estimated by a regression method in SAL model |
| P/B | CAL 5.240  SAL 5.240 | Buia and Marzocchi, 1995, Banaru et al., 2013; Agnetta et al., 2019 |  |
| 54. L phytoplank |  |  | Large phytoplankton. |
| Bi (t km^-1^ y^-^) | CAL 3.0682  SAL 3.0682 | Lazzari et al., 2012 | Model OPATM-BFM |
| P/B | CAL 175.785  SAL 175.785 | Lazzari et al., 2012; Agnetta et al., 2019 | Model OPATM-BFM |
| 55. S phytoplank |  |  | Small phytoplankton. |
| Bi (t km^-1^ y^-^) | CAL 2.8936  SAL 2.8936 | Lazzari et al., 2012 | Model OPATM-BFM |
| P/B | CAL 205.620  SAL 205.620 | Lazzari et al., 2012; Agnetta et al., 2019 | Model OPATM-BFM |
| 56. MS |  |  |  |
| Bi (t km^-1^ y^-^) | - |  | Default value of model 1.000 |
| 57. Disc |  |  |  |
| Bi (t km^-2^ y^-1^) | - |  | Default value of model 1.000 |
| 58. Det |  |  |  |
| Bi (t km^-2^ y^-1^) | CAL 1.940  SAL 1.690 | Pauly et al., 1993 | Detritus biomass was estimated from primary production using an empirical equation (Pauly et al., 1993). |

FG = Functional group; M= Natural mortality; Z= total mortality; Von Bertalanffy Growth Equation parameters and L-W relationships’ coefficients required for M and Q/B estimations when applying empirical relationships (Pauly, 1980; Pauly et al., 1990) were taken from D’Onghia et al. (2000, 2006), Froese and Pauly (2008), Carlucci et al. (2009), Maiorano et al. (2010) or from MEDITS data analysis of GSA 19.

**References listed in Table S1**

Abelló, P., 1989. Feeding habits of *Macropipus tuberculatus* (Brachyura, Portunidae) off the Catalan coast (NW Mediterranean). Misc. Zool., 13: 45-50.

Ainsworth, C., Heymans, J.J., Pitcher, T., Vasconcellos, M., 2002. Ecosystem models of Northern British Columbia for the time periods 2000, 1950, 1900 and 1750. Fisheries Centre Research Reports, 10(4): 41 pp.

Anastasopoulou, A., Kapiris, K., 2008. Feeding ecology of the shortnose greeneye *Chlorophthalmus agassizi* Bonaparte, 1840 (Pisces: Chlorophthalmidae) in the eastern Ionian Sea (eastern Mediterranean). Journal of Applied Ichthyology 24, 170-179.

Arreguín-Sánchez, F., Arcos, E., Chávez, E.A., 2002. Flows of biomass and structure in an exploited benthic ecosystem in the Gulf of California, Mexico. Ecol. Model. 156, 167–183.

Bacha, M., Moali, A., Benmansour, N.E., Brylinski, J.M., Mahé, K., Amara, R., 2010. Relationships between age, growth, diet and environmental parameters for anchovy (*Engraulis encrasicolus* L.) in the Bay of Bénisaf (SW Mediterranean, west Algerian coast). Cybium, 34(1): 47-57.

Başçınar, N.S., Sağlam, H., 2009. Feeding Habits of Black Scorpionfish *Scorpaena porcus*, in the South-Eastern Black Sea. Turk. J. Fish. Aquat. Sci., 9: 99-103.

Battaglia, P., Andaloro, F., Consoli, P., Esposito, V., Malara, D., Musolino, S., Peda`, C., Romeo, T., 2013. Feeding habits of the Atlantic bluefin tuna, *Thunnus thynnus* (L. 1758), in the central Mediterranean Sea (Strait of Messina). Helgol Mar Res., 67: 97–107.

Bell, J.D., Harmelin-Vivien, M.L., 1983. Fish fauna of French Mediterranean *Posidonia oceanica* seagrass meadows. 2. Feeding habits. Tethys 11, 1-14.

Bello, G., 1991. Role of Cephalopods in the Diet of the Swordfish, *Xiphias gladius*, from the Eastern Mediterranean Sea. Bulletin of Marine Science, 49(1-2): 312-324.

Belluscio, A., U. Scacco, F. Colloca, P. Carpentiere and G.D. Ardizzone, 2000. Feeding strategies of two species of demersal Chondrichthyans, *Galeus melastomus* (Rafinesque, 1810) and *Etmopterus spinax* (Linnaeua, 1758), in the Central Tyrrhenian Sea. Biol. Mar. Mediterr. 7(1):417-426.

Bergstad, O.A., 1991. Distribution and trophic ecology of some gadoid fish of the Norwegian Deep. 1. Accounts of individual species. Sarsia, 75: 269-313.

Bergstrom, O., 1985. Aspects of natural foraging by *Sepietta oweniana* (Mollusca, Cepalophoda). Ophelia, 24(1): 65-74 .

Bernardez, C., Freire, J., Gonzalez-Gurriaran, E., 2000. Feeding of the spider crab *Maja squinado* in rocky subtidal areas of the Ria de Arousa (north-west Spain). Journal of the Marine Biological Association of the UK 80, 95-102.

Beverton R.J.H. and Holt S.J., 1957. On the dynamics of exploited fish populations. Fish. Invest. Minist. Agric. Fish. Food G.B. Ser. II 19.533p.

Blanco, C., Salomón, O., Raga, J.A., 2001. Diet of the bottlenose dolphin (*Tursiops truncatus*) in the Western Mediterranean Sea. Journal of the Marine Biological Association of the UK 81, 1053-1058.

Bloch, D., Desportes, G., Harvey, P., Lockyer, C., Mikkelsen, B., 2012. Life History of Risso’s Dolphin (*Grampus griseus*) (G. Cuvier, 1812) in the Faroe Islands. Aquatic Mammals, 38(3): 250-266.

Bozzano, A., Recasens, L., Sartor, P., 1997. Diet of the european hake *Merluccius merluccius* (Pisces: Merluciidae) in the Western Mediterranean (Gulf of Lions). Scientia Marina 61: 1-8.

Brey T., 2001. Population dynamics in benthic invertebrates. A virtual handbook. Version 01.2. http://www.thomas-brey.de/science/virtualhandbook.

Bradai, M.N., Bouain, A., 1990. Régime alimentaire de *Scorpaena porcus* et de *S. scrofa* (Teleostei, Scorpaenidae) du Golfe de Gabès, Tunisie. Cybium 14: 207-216.

Brando V.A, Ceccarelli R., Libralato S., Ravagnan G., 2004. Assessment of environmental management effects in a shallow water basin using mass-balance models. Ecological Modelling, 172: 213–232.

Cabral, E.N., Lopes, M., Loeper, R., 2002. Trophic niche overlap between flatfihes in a nursery area on the Portuguese coast. Sci. Mar., 66(3): 293-300.

Cabral, H.N., Murta, A.G., 2002. The diet of blue whiting, hake, horse mackerel and mackerel off Portugal. Journal of Applied Ichthyology 18, 14-23.

Cammen L.M., 1980. Ingestion rate: an empirical model for aquatic deposit feeders and detritovores. Oecologia, 44: 303-310

Campo, D., Mostarda, E., Castriota, L., Scarabello, M.P., Andaloro, F., 2006 Feeding habits of the Atlantic bonito, *Sarda sarda* (Bloch, 1793) in the southern Tyrrhenian sea. Fisheries Research 81, 169-175.

Cardinale, M., F. Colloca and Ardizzone, G.D., 1997. Feeding ecology of Mediterranean razorfish *Xyrichthys novacula* in the Tyrrhenian Sea (Central Mediterranean Sea). J. Appl. Ichthyol., 13(3):105-111.

Carlucci, R., Capezzuto, F., Maiorano, P., Sion, L., D’Onghia, G. 2009. Distribution, population structure and dynamics of the black anglerfish (*Lophius budegassa*) (Spinola, 1987) in the Eastern Mediterranean Sea. Fisheries Research 95 (2009) 76–87

Carpentieri, P., Colloca, F. and Ardizzone, G. (2007), Rhythms of feeding activity and food consumption of two Mediterranean burrowing fishes: *Gnathophis mystax* (Delaroche) and *Chlopsis bicolor* Rafinesque. Marine Ecology, 28: 487–495.

Carpentieri, P., Colloca, F., Belluscio, A., Criscoli, A., Ardizzone, G.D., 2006. Diel feeding periodicity and daily ration of shelf break species. Journal of the Marine Biological Association of the UK, 86(04): 853 - 860.

Carrassón, M., Matallanas, J., 2002. Diets of deep-sea macrourid fishes in the western Mediterranean. Mar. Ecol. Prog. Ser., 234: 215–228.

Carrassón, M., Matallanas, J., Casadevall, M., 1997. Feeding strategies of deep-water morids on the western Mediterranean slope. Deep-Sea Research I, 44: 9-10.

Carrassón, M., Stefanescu, C. and Cartes, J.E., 1992. Diets and bathymetric distributions of two bathyal sharks of the Catalan deep sea (western Mediterranean). Mar. Ecol. Prog. Ser., 82: 21–30

Cartes, J.E., 1995. Diets of, and trophic resources exploited by, bathyal penaeoidean shrimps from the western Mediterranean. Mar. Freshwater Res., 46: 889-96.

Cartes, J.E., Abello, P., 1992. Feeding of Mediterranean polychelid lobsters. Mar. Ecol. Prog. Ser., 84: 139-150.

Cartes, J.E., 1993a. Diets of deep-water pandalid shrimps on the Western Mediterranean Slope. Marine Ecology Progress Series 96, 49-61.

Cartes, J.E., 1993b. Feeding habits of pasiphaeid shrimps close to the bottom on the Western Mediterranean slope. Marine Biology, 117: 459-468.

Cartes, J.E., 1993c. Diets of deep-sea brachyuran crabs in the Western Mediterranean Sea. Marine Biology 117, 449-457

Cartes, J.E., 1993d. Diets of two deep-sea decapdos: *Nematocarcinus exilis* (caridea: nematocarcinidae) and *Munida tenuimana* (anomura: galatheidae) on the western mediterranean slope. Ophelia, 37 (3): 213-229.

Casadevall, M., Matallanas, J., Bartolν, T., 1994. Feeding habits of *Ophichthus rufus* (Anguilliformes, Ophichthidae) in the western Mediterranean. Cybium 18, 431-440.

Casadevall, M., Matallanas, J., 1990. Feeding habits of *Gnathophis mystax* (Delaroche, 1809), (Anguilliformes, Congridae) in the western Mediterranean. Journal of Fish Biology 37, 827-829.

Casale, P., Abbate, G., Freggi, D., Conte, N., Oliverio, M., Argano, R., 2008. Foraging ecology of loggerhead sea turtles *Caretta caretta* in the central Mediterranean Sea: evidence for a relaxed life history model. Mar. Ecol. Prog. Ser., 372: 265–276.

Casale, P., Mazaris, A.D., Freggi, D., Basso, R., Argano, R., 2007. Survival probabilities of loggerhead sea turtles (*Caretta caretta*) estimated from capture-mark-recapture data in the Mediterranean Sea. Sci. Mar., 71(2): 365-372.

Castro, J.J., Hernandez-Garcìa, V., 1995. Ontogenetic changes in mouth structures, foraging behaviour and habitat use of *Scomber japonicus* and *Illex coindetii*. Sci. Mar., 59(3-4): 347-355.

Castro, B.G., Guerra, A., 1990. The diet of *Sepia officinalis* (Linnaeus, 1758) and *Sepia elegans* (D'Orbigny, 1835) (Cephalopoda, Sepioidea) from the Ría de Vigo (NW Spain). Scientia Marina 54, 375-388.

Childress, J. J., Taylor, S.M., Cailliet, G. M., and Price, M. H. 1980. Patterns of growth, energy utilization and reproduction in some meso- and bathypelagic fishes off southern California. Mar. Bioi., 61:27-40.

Coelho, M., Domingues, P., Balguerias, E., Fernandez, M., Andrade, J.P., 1996. A comparative study of the diet of *Loligo vulgaris* ( Lamarck, 1799) ( Mollusca:Cephalopoda) from the south coast of Portugal and the Saharan Bank (Central-East Atlantic). Fisheries Research, 29(I 997): 245-255.

Coll, M., Palomera, I., Tudela, S., Dowd, M., 2008. Food-web dynamics in the South Catalan Sea ecosystem (NW Mediterranean) for 1978–2003. Ecol. Model., 217(1–2): 95–116

Coll, M., Santojanni, A., Palomera, I., Tudela, S., Arneri, E., 2007. An ecosystem model of the Northern and Central Adriatic Sea: analysis of ecosystem structure and ﬁshing impacts. J. Mar. Syst. ; 67: 119–154.

Coll, M., Palomera, I., Tudela, S., Sarda, F., 2006. Trophic flows, ecosystem structure and fishing impacts in the South Catalan Sea, Northwestern Mediterranean. Journal of Marine Systems 59 63- 96.

Colloca, F., Cardinale, M. and Ardizzone, G.D. 1997. Biology, spatial distribution and population dynamics of *Lepidotrigla cavillone* (Pisces: Triglidae) in the Central Tyrrhenian Sea. Fish. Res. 32:21-32.

Consoli, P., Battaglia, P., Castriota, L., Esposito, V., Romeo, T., Andaloro, F., 2010. Age, growth and feeding habits of the bluemouth rockfish, *Helicolenus dactylopterus dactylopterus* (Delaroche 1809) in the central Mediterranean (southern Tyrrhenian Sea). J. Appl. Ichthyol., 26: 583–591.

Cortes, E., 1999. Standardized diet compositions and trophic levels of sharks. ICES Journal of Marine Science 56, 707-717.

Corrales X., Coll M., Tecchio S., Bellido J.M., Fernàndez A.M., Palomera I., 2015. Ecosystem structure and fishing impacts in the northwestern Mediterranean Sea using a food web model within a comparative approach. Journal of Marine Systems, 148: 183-199

Cristo, M. , Cartes, J.E., 1998. A comparative study of the feeding ecology of *Nephrops norvegicus* L. (Decapoda: Nephropidae) in the bathyal Mediterranean and the adjacent Atlantic. Scientia Marina 62 (Suppl. 1), 81-90

D’Onghia G., Sion L., Maiorano P., Mytilineou C., Dalessandro S., Carlucci R., Desantis S., 2006. Population biology and life strategies of *Chlorophthalmus agassizii* Bonaparte, 1840 (Pisces: Osteichthyes) in the Mediterranean Sea. Marine Biology, 149: 435-446.

D’Onghia, G., Mastrototaro, F., Maiorano, P., 2000. Biology of silver scabbard fish, *Lepidopus caudatus* (Trichiuridae), from the Ionian Sea (Eastern-Central Mediterranean). Cybium, 24(3):249-262.

Evans, K., Hindell, M.A., 2004. The diet of sperm whales (Physeter macrocephalus) in southern Australian waters. ICES Journal of Marine Science, 61: 1313-1329.

Fanelli E., Badalamenti F., D’Anna, G., Pipitone, C., Riginella, E. Azzurro, E., 2011. Food partitioning and diet temporal variation in two coexisting sparids, *Pagellus erythrinus* and *Pagellus acarne*. J. Of Fish Biology, 78: 869-900.

Fanelli E., Badalamenti, F., D’anna, G., Pipitone, C., 2009. Diet and trophic level of scaldfish *Arnoglossus laterna* in the southern Tyrrhenian Sea (western Mediterranean): trasting trawled versus untrawled areas. Journal of the Marine Biological Association of the UK, 89(4): 817–828.

Fanelli, E., Cartes, J.E., 2004. Feeding habits of pandalid shrimps in the Alboran Sea (SW Mediterranean): influence of biological and environmental factors. Mar. Ecol. Prog. Ser., 280: 227–238.

Fasola, M., Bogliani, G., Saino, N., Canova., L., 1989. Foraging, feeding and time-activity niches of eight species of breeding seabirds in the coastal wetlands of the Adriatic Sea. Boll. Zool., 56: 61-72.

Fauchald, K., Jumars, P.A., 1979. The diet of worms: a study of Polychaete feeding guilds. Oceanogr. Mar. Biol. Ann. Rev ., 17: 193-284.

Corrales X, Coll M., Tecchio S., Bellido J.M., Fernández A.M., Palomera I., 2015. Ecosystem structure and fishing impacts in the northwestern Mediterranean Sea using a food web model within a comparative approach. Journal of Marine Systems, 148: 183–199.

Freire, J., 1996. Feeding ecology of *Liocarinus depurator* (Decapoda: Portunidae) in the Ría de Arousa (Galicia, north-western Spain): effects of habitat, season and life history. Marine Biology 126, 297-311.

Froese, R.J., Pauly, D. 2008. Fishbase. World wide web electronic publication. Available from <http://www.fishbase.org>.

Gorelova, T.A. Krasil'nikova, N.A., 1990. On the diet of *Maurolicus muelleri* in the vicinity of Seamounts Discovery, Nasca, and Mt. Africana. J. Ichthyol., 30(7): 42-52.

Guénette, S., Morato, T., 2001. The Azores Archipelago, 1997, p. 241-270. In: S. Guénette, V.Christensen and D. Pauly (Eds.), Fisheries impacts on North Atlantic Ecosystems: Models and analyses. Fisheries Centre Research Reports 9(4). University of British Columbia, Vancouver, BC, Canada. [Available online from www.fisheries.ubc.ca].

Guerra, A., Rocha, F., 1994. The life history of *Loligo vulgaris* and *Loligo forbesi* (Cephalopoda: Loliginidae) in Galician waters (NW Spain). Fisheries Research, 21(1-2): 43-69.

Halpin, P.N., A.J. Read, E. Fujioka, B.D. Best, B. Donnelly, L.J. Hazen, C. Kot, K. Urian, E. LaBrecque, A. Dimatteo, J. Cleary, C. Good, L.B. Crowder, and K.D. Hyrenbach. 2009. OBIS-SEAMAP: The world data center for marine mammal, sea bird and sea turtle distributions. Oceanography, 22(2): 104-115.

Harmelin-Vivien, M.L., Kaim-Malka, R.A., Ledoyer, M., Jacob-Abraham, S.S., 1989. Food partitioning among scorpaenid fishes in Mediterranean seagrass beds. Journal of Fish Biology 34, 715-734

Hattab T., Frida Ben Rais Lasram , Camille Albouy, Mohamed Salah Romdhane, Othman Jarboui, Ghassen Halouani, Philippe Cury, François Le Loc'h, (2013) An ecosystem model of an exploited southern Mediterranean shelf region (Gulf of Gabes, Tunisia) and a comparison with other Mediterranean ecosystem model properties. Journal of Marine Systems 128 159–174.

Heymans, S. 2005. Ecosystem models of the western and central Aleutian Islands in 1963, 1979 and 1991. In Foodweb models and data for studying fisheries and environmental impact on Eastern Pacific ecosystems. Edited by S. Guénette and V. Christensen. Fisheries Centre, The University of British Columbia, Vancouver, B.C. Fish. Cent. Res. Rep. Vol. 13(1). pp. 8–82.

Hopkins, T.L., Sutton, T.T, Lancraft, T.M., 1996. The trophic structure and predation impact of a low latitude midwater fish assemblage. Prog. Oceanog., 38: 205-239.

Hopkins, T.L., Baird, R.C., 1985. Feeding ecology of four hatchetfishes (Sternoptychidae) in the eastern Gulf of Mexico. Bulletin of Marine Science 36, 260-277.

Jardas, I., Šantić, M., Pallaoro, A., 2004. Diet composition and feeding intensity of horse mackerel, *Trachurus trachurus* (Osteichthyes: Carangidae) in the eastern Adriatic. Marine Biology, 144: 1051–1056;

Juki, S., 1972. Nutrition of the hake (*Merluccius merluccius*), bogue (*Boops boops*), striped mullet (*Mullus barbatus*) and pandora (*Pagellus erythrinus*) in the Bay of KaÅ¡tela. Acta Adriatica, 14: 3-40.

Kapiris, K., Thessalou-Legaki M., 2011. Feeding ecology of the deep-water blue–red shrimp *Aristeus antennatus* (Decapoda: Aristeidae) in the Greek Ionian Sea (E. Mediterranean). J. Sea Res., 65: 151-160.

Kapiris, K., 2004. Feeding ecology of *Parapenaeus longirostris* (Lucas, 1846) (Decapoda: Penaeidae) from the Ionian Sea (Central and Eastern Mediterranean Sea). Scientia Marina 68, 247-256.

Karakulak, F.S., Salman, A. and Oray, I.K., 2009. Diet composition of bluefin tuna (*Thunnus thynnus* L. 1758) in the Eastern Mediterranean Sea, Turkey. J. Appl. Ichthyol., 25: 757–761.

Khoury, C., 1984. Ethologies alimentaires de quelques espèces de poisons de l'herbier de Posidonies du Parc National de Port-Cros. In Boudouresque C.F., Jeudy de Grissac A. and Olivier J. (eds.) International Workshop Posidonia Oceanica Beds, GIS Posidonie Publications, France 1, 335-347.

Kyrtatos, N.A., 1982. Investigation on fishing and biology of the most important fishes of the region around the Aegean Sea. Island of Tinos. Thalassographica 5 (specl. publ.), 88 pp.

Labropoulou, M., Eleftheriou, A., 1997. The foraging ecology of two pairs of congeneric demersal fish species: importance of morphological characteristics in prey selection. Journal of Fish Biology 50, 324-340.

Labropoulou, M., Machias, A., 1998. Effect of habitat selection on the dietary patterns of two triglid species. Marine Ecology Progress Series 173, 275-288.

Laran, S., Joiris, C., Gannier, A., Kenney, R.D., 2010. Seasonal estimates of densities and predation rates of cetaceans in the Ligurian Sea, northwestern Mediterranean Sea: an initial examination. J. Cetacean Res. Manage., 11(1): 31-40.

Lazzari, P., Solidoro, C., Ibello, V., Salon, S., Teruzzi, A., Béranger, K., Colella, S., Crise, A., 2012. Seasonal and inter-annual variability of plankton chlorophyll and primary production in the Mediterranean Sea: a modelling approach. Biogeosciences, 9: 217-233.

Longo C., Colloca F., Carpentieri P., Belluscio A., Ardizzone G.D., 2005. Strategie addattative e segregazione trofica tra *Argentina sphyraena* e *Glossanodon leioglossus* (Teleostea, Argentinidae). Biol. Mar. Medit., 12(1): 540-543.

Lopez S., 2013. L’ecosistema del Mar Tirreno: aspetti strutturali, funzionali, effetti della pesca e delle interazioni trofiche. PhD Thesis, University of Rome "La Sapienza". http://hdl.handle.net/11573/768393;

Mackinson, S., Okey, T., Vasconcellos, M., Vidal-Hernandez, L.,Mahmoudi, B. (Eds.), (2000). An ecosystem model of the West Florida Shelf for use in fisheries management and ecological research. Technical report. Florida Fish and Wildlife Conservation Commission, Florida Marine Research Institute, St. Petersburg.

Macpherson, E., 1981. Resource Partitioning in a Mediterranean Demersal Fish Community. Marine Ecology Progress Series 4, 183-193.

Macpherson, E., 1979. Relations trophiques des poisons dans la Méditerranée occidentale. Rapp. Comm. Int. Explor. Sci. Mer Méditerr., 25/26: 49-58.

Madurell, T., Cartes, J.E., 2005. Trophodynamics of a deep-sea demersal fish assemblage from the bathyal eastern Ionian Sea (Mediterranean Sea). Deep-Sea Research I 52, 2049-2064.

Maiorano, P., Sion, L., Carlucci, R., Capezzuto, F., Giove, A., Costantino, G., … Tursi, A. (2010). The demersal faunal assemblage of the NW Ionian Sea (Central Mediterranean): current knowledge and perspectives. Chemistry and Ecology, 26, 219–240.

Malej, A., 1989. Behaviour and trophic ecology of the jellyf ish *Pelagia noctiluca* (Forsskaal, 1775). Journal of Experimental Marine Biology and Ecology 126 (3), 259–270.

Maynou F. and Cartes J.E., 1998. Daily ration estimates and comparativc study of food consumption in nine species of deep-water decapod crustaceans of the NW Mediterranean. Mar Ecol Prog Ser, 171 :221-231

Megalofonu, P., Chatzispyrou, A., 2006. Sexual maturity and feeding of the gulper shark, *Centrophorus granulosus*, from the eastern Mediterranean Sea. Cybium, 30(4): 67-74.

Merz, G., Myers, R.A., 1998. A simplified formulation for fish production. Can. J. Fisheries Aquatic Sci. 55 (2), 478– 484.

Meyer, M., Smale, M.J., 1991. Predation patterns of demersal teleosts from the Cape south and west coasts of South Africa.1. Pelagic predators. S. Afr. J. mar. Sci., 11: 173-191.

Morato, T., Solà, E., Grós, M.P., Menezes, G., 2001. Feeding habits of two congener species fo seabreams, Pagellus bogaraveo and P. acarne, off the Azores (Northeastern Atlantic) during spring of 1996 and 1997. Bull. Mar. Sci., 69(3): 1073-1087.

Morato, T., Solà, E., Grós, M.P., Menezes, G., 1999. Diets of forkbeard (*Phycis phycis*) and conger eel (Conger conger) off the Azores during spring of 1996 and 1997. Life Mar. Sci., 17A: 51-64.

Moreno-Amich, R., 1992. Feeding habits of red gurnard, *Aspitrigla cuculus* (L. 1758) (Scorpaeniformes, Triglidae), along the Catalan coast (northwestern Mediterranean). Hydrobiologia 228, 175-184.

Moreno-Amich, R., 1994. Feeding habits of grey gurnard, *Eutrigla gurnardus* (L., 1758), along the Catalan coast (northwestern Mediterranean). Hydrobiologia 273, 57-66.

Morte, M.S, Redon, M.J., Sanz-Brau, A., 1997. Feeding habits of juvenile *Mustelus mustelus* (Carcharhiniformes, Triakidae) in the western Mediterranean. Cah. Biol. Mar., 38:103-107.

Morte M.S., Redòn, M.J., Sanz-Brau, A., 2002. Diet of *Phycis blennoides* in relation to Fish Size and Season in the Western Mediterranean (Spain). Marine Ecology, 23 (2): 141-155.

Morte, S., Redon, M.J., Sanz-Brau, A., 1999. Feeding ecology of two megrims *Lepidorhombus boscii* and *Lepidorhombus whiffiagonis* in the western Mediterranean (Gulf of Valencia, Spain). Journal of the Marine Biological Association of the UK 79, 161-169.

Moutopoulos, D.K., Libralato, S., Solidoro, C. & Stergiou, K.I. (2013). Toward an ecosystem approach to fisheries in the Mediterranean Sea: Multi-gear/multi-species implications from an ecosystem model of the Greek Ionian Sea. Journal of Marine Systems, 113–114, 13–28.

Nagy, K.A., 1878. Field methabolic rate and food requirement scaling in mammals and birds. Ecol. Monogr., 57: 111-127.

O'Sullivan, S., Moriarity, C., Davenport, J., 2004. Analysis of the stomach contents of the European conger eel *Conger conger* in Irish waters. J. Mar. Biol. Ass. U.K., 84(4): 823-826.

Olaso, I. Rodriguez-Marin, E., 1995. Alimentación de veinte especies de peces demersales pertenecientes a la división VIIIc del ICES. Otoño 1991. Informes Técnicos, Centro Oceanográfico de Santander, Instituto Español de Oceanografía, 56p.

Pakhomov, E.A.; Perissinotto, R.; McQuaid, C.D.I, 1996: Prey composition and daily rations of myctophid fishes in the Southern Ocean. Marine Ecology Progress Series, 134: 1-14

Pauly, D., Trites, A.W., Capuli, E. and Christensen, V., 1998. Diet composition and trophic levels of marine mammals. ICES Journal of Marine Science, 55: 467–481.

Pauly, D., 1980. On the interrelationships between natural mortality, growth parameters, and mean environmental temperature in 175 fish stocks. Journal du Conseil, Conseil International pour l’Exploration de la Mer 39, 175-192.

Pauly, D., Christensen, V., Sambilay, V., 1990. Some features of fish food consumption estimates used by ecosystem modellers. ICES CM/G:17, 8 pp.

Piroddi, C., Coll, M., Steenbeek, J., Moy, D.M. and Christensen, V. (2015) Modelling the Mediterranean marine ecosystem as a whole: Addressing the challenge of complexity. Marine Ecology Progress Series 533, 47–65.

Plounevez S, Champalbert G (2000) Diet, feeding behavior and trophic activity of the anchovy (*Engraulis encrasicolus* L.) in the Gulf of Lions (Mediterranean Sea). Oceanol Acta, 23: 175-192.

Podrazhanskaya, S.G., 1993. Feeding Habits of Mesopelagic Species of Fish and Estimation of Plankton Graze in the Northwest Atlantic. NAFO Sci. Coun. Studies, 19: 79–85.

Quetglas, A., Alemany, F., Carbonell, A., Merella, P., Sánchez, P., 1998. Biology and fishery of *Octopus vulgaris* Cuvier, 1797, caught by trawlers in Mallorca (Balearic Sea, Western Mediterranean). Fisheries Research 36, 237-249.

Quetglas, A., de Mesa, A., Ordines, F., Grau, A., 2010. Life history of the deep-sea cephalopod family Histioteuthidae in the western Mediterranean. Deep-Sea Research I, 57: 999–1008;

Quetglas A., Ordines F., Gonzalez M., Franco I., 2009. Life history of the bathyal octopus *Pteroctopus tetracirrhus* (Mollusca, Cephalopoda) in the Mediterranean Sea. Deep-Sea Research I, 56: 1379–1390.

Quetglas, A., Gonzalez, M., Franco, I., 2005. Biology of the upper-slope cephalopod *Octopus salutii* from the western Mediterranean Sea. Marine Biology, 146: 1131–1138.

Quetglas, A., Alemany, F., Carbonell, A., Merella, P., Sánchez, P., 1999. Diet of the European flying squid *Todarodes sagittatus* (Cephalopoda: Ommastrephidae) in the Balearic Sea (western Mediterranean). Journal of the Marine Biological Association of the UK 79, 479-486.

Rasero, M., Gonzalez, A.F., Castro, B.G., Guerra, A., 1996. Predatory relationships of two sympatric squid, *Todaropsis eblanae* and *Illex coindetii* (Cephalopoda: ommastrephidae) in Galician waters. Journal of the Marine Biological Association of the UK 76, 73-87.

Relini, G., 2002. Trophic relationships between fishes and an artificial reef. ICES Journal of Marine Science, 59: S36–S42.

Ristow, D., Feldmann, F., Scharlau, W., Wink, M., 1990. Population structure, phylopatry and mortality of Cory’s Shearwater Clonectris d. diomedea. Vogelwelt, 111: 172-181.

Romanelli, M., Consalvo, I., Vacchi, M., Finoia, M.G., 2006. Diet of *Torpedo torpedo* and *Torpedo marmorata* in a coastal area of Central Western Italy (Mediterranean Sea). Mar. Life, 16: 21-30.

Rosa, R., Marques, A.M., Nunes, M.L., Bandarra, N., Reis, C.S., 2004. Spatial-temporal changes in dymetil acetal (octadecanal) levels of *Octopus vulgaris* (Cephalopoda): relation to feeding ecology. Sci. Mar., 68 (2): 227-236

Rosecchi, E., 1987. L’ alimentation de *Diplodus annularis*, *Diplodus sargus*, *Diplodus vulgaris* et *Sparus aurata* (Pisces, Sparidae) dans le Golfe du Lion et les lagunes littorales. Revue des Travaux de l'Institut des Peches Maritimes 49, 125-141.

Sala, E., Ballesteros, E., 1997. Partitioning of space and food resources by three fish genus Diplodus (Sparidae) in a Mediterranean rocky infralittoral ecosystem. Marine Ecology Progress Series 152, 273-283.

Samir, I., 2008. Feeding habits of the Atlantic stargazer fish *Uranoscopus scaber* Linnaeus, 1758 (Family: Uranoscopidae) in Egyptian Mediterranean waters. Egypt. J.AquaL Biol. & Fish, 12: 1-11.

Šantić, M., 2010. Diet of striped sea bream *Lithognathus mormyrus* (Sparidae) from eastern central Adriatic Sea. Cybium, 34(4): 345-352.

Šantić, M., Jardas, I., Pallaoro, A., 2003. Feeding habits of Mediterranean horse mackerel, *Trachurus mediterraneus* (Carangidae), in the central Adriatic Sea. Cybium, 27(4): 247-253.

Šantić, M., Jardas, I., Pallaoro, A., 2005. Feeding habits of horse mackerel, *Trachurus trachurus* (Linneaus, 1758), from the central Adriatic Sea. Journal of Applied Ichthyology 21, 125-130.

Sanz, A., 1985. Contribución al estudio de la biología de *Uranoscopus scaber* Linnaeus, 1758 (Osteichthyes, Uranoscopidae) del Mediterráneo occidental. Investigación pesquera 49, 35-46.

Sever, T.M., Bayhan, B., Taskavak, E., 2005 A Preliminary Study on the Feeding Regime of European Pilchard (*Sardina pilchardus* Walbaum1792) in Izmir Bay, Turkey, Eastern Aegean Sea. Naga - International Center for Living Aquatic Resources Management 28, 41-48.

Sever, T.M., Bayhan, B., Bilecenoglu, M., Mavili, S., 2006. Diet composition of the juvenile chub mackerel (*Scomber japonicus*) in the Aegean Sea (Izmir Bay, Turkey). Journal of Applied Ichthyology 22, 145-148.

Sifner, S.K., Vrgoc, N., 2009. Diet and feeding of the musky octopus, *Eledone moschata*, in the northern Adriatic Sea. Journal of the Marine biological Association of the UK, 89(2): 413-419.

Sirotenko, M.D., Sorokalit, L.K., 1979. Seasonal changes in the food of the Mediterranean sprat,*Sprattus sprattus* *phalericus*. J. Ichthyol., 19(5): 37-51.

Smale, M.J., 1996. Cephalophods as prey. IV. Fishes. Phil. Trans. R. Soc. Lond. B., 351: 1067-1081.

Soares, M.S.C., Sousa, L., Barreiros, J.P., 2003. Feeding habits of the lizardfish *Synodus saurus* (Linnaeus, 1758) (Actinopterygii: Synodontidae) from the Azores. Aqua, J. Ichthyol. Aquat. Biol., 7(1): 29-38.

Spitz, J., Richard, E., Meynier, L., Pusineri, C., Ridoux, V., 2006. Dietary plasticity of the oceanic striped dolphin, *Stenella coeruleoalba*, in the neritic waters of the Bay of Biscay. Journal of Sea Research, 55: 309–320.

Stagioni, M., 2013. Feeding habits of anglerﬁsh, *Lophius budegassa* (Spinola, 1807) in the Adriatic Sea, north-eastern Mediterranean. J. Appl. Ichthyol., 29: 374–380.

Stagioni, M., 2012. Feeding of tub gurnard *Chelidonichthys lucerna* (Scorpaeniformes: Triglidae) in the north-east Mediterranean. Journal of the Marine Biological Association of the United Kingdom, 92(3): 605–612.

Stagioni, M., Montanini, S., Vallisneri, M., 2011. Feeding Habits Of European Hake, *Merluccius Merluccius* (Actinopterygii: Gadiformes: Merlucciidae), From The Northeastern Mediterranean Sea. Acta Ichthyologica et Piscatoria, 41(4): 277–284.

STECF (Scientific, Technical and Economic Committee for Fisheries), 2011. Assessment of Mediterranean Sea stocks - part 1 (STECF-11-08). Edited by Alvaro Abella, Hans- oachim R tz and Aymen Charef.

Stefanescu, C., Cartes, J.E., 1992. Benthopelagic habits of adult specimens of *Lampanyctus crocodilus* (Risso 1810) (Osteichthyes, Myctophidae) in the western Mediterranean deep slope. Sci. Mar., 56(1): 69-74.

Stergiou, K.I., 1993. Abundance-depth relationship, condition factor and adaptive value of zooplanktophagy of red bandfish, *Cepola macrophthalma*. Journal of Fish Biology 42, 645-660

Stergiou, K.I., Fourtouni, H., 1991. Food habits, ontogenetic diet shift and selectivity in *Zeus faber* Linnaeus, 1758. Journal of Fish Biology 39, 589-603.

Terrats, A., Petrakis, G., Papacostantinou, C., 1999. Feeding habits of *Aspitrigla cuculus* (L., 1758) (red gurnard), *Lepidotrigla cavillone* (Lac., 1802) (large scale gurnard) and *Trigloporus lastoviza* (Brunn., 1768) (rock gurnard) around Cyclades and Dodecanese Islands (E. Mediterranean). Mediterranean Marine Science, 1/1: 91-104.

Tomas, J., Aznar, F.J., Raga, J.A., 2001. Feeding ecology of the loggerhead turtle *Caretta caretta* in the western Mediterranean. Journal of Zoology 255, 525-532.

Trites, A.W., Pauly, D., 1998. Estimating mean body masses of marine mammals from maximum body lengths. Can. J. Zool., 76: 886–896.

Tsarin, S.A., 1994. Age, growth, and some production characteristics of *Ceratoscopelus warmingii* (Myctophidae) in the tropical zone of the Indian Ocean. J. Ichthyol. 34(6):59-72.

Tudela S, Palomera I (1995) Diel feeding intensity and daily ration in the anchovy *Engraulis encrasicolus* in the northwest Mediterranean Sea during the spawning period. Mar Ecol Prog Ser 129:55-61

Tuncay, M.S., Bahar, B., Semih, L., 2010. Feeding habits of *Cepola macrophthalma* (pisces: cepolidae) in izmir bay, Aegean Sea. Rapp. Comm. int. Mer Médit., 39: 598. CIESM

Tuncay, M.S, Halit, F., Bahar B., Ertan T., Gökçen B., 2008. Food habits of the hollowsnout grenadier, *Caelorinchus caelorhincus* (Risso, 1810), in the Aegean Sea, Turkey. Belg. J. Zool., 138(1): 81-84.

Vannucci S., Mancusi C., Serena F., Cuoco C., Voliani A:, 2006. Feeding ecology of rays in Ligurian Sea. Biol. Mar. Medit., 13(2): 296-297.

Vassilopoulou, V., 2006. Dietary habits of the deep-sea flatfish *Lepidorhombus boscii* in north-eastern Mediterranean waters. Journal of Fish Biology 69, 1202-1220.

Velasco, F., Olaso, I., de la Gándara, F., 1996. Alimentación de veintidós especies de peces demersales de la División VIIIc de la ICES. Otoños de 1992 y 1993. Informes Técnicos, Instituto Español de Oceanografía 164: 62.

Wells, J.M. & Clarke, A., 1996. Energetics: the cost of living and reproducing for an individual Cephalopod. Philosophical Transactions: Biological Sciences, 351(1343): 1083-1104

Würtz, M., Marrale, D., 1993. Food of striped dolphin, *Stenella coeruleoalba*, in the Ligurian Sea. Journal of the Marine Biological Association of the UK 73, 571-578.

Yeldan, H., Avsar, D., Manasırlı, M., 2009. Age, growth and feeding of the common stingray (*Dasyatis pastinaca*, L., 1758) in the Cilician coastal basin, northeastern Mediterranean Sea. J. Appl. Ichthyol., 25(1):, 98–102

Yıgın, C., Ismen, A., 2010. Age, growth, reproduction and feed of longnosed skate, *Dipturus oxyrinchus* (Linnaeus, 1758) in Saros Bay, the north Aegean Sea. J. Appl. Ichthyol., 26: 913–919.

Table S2. Input parameters of the CAL and SAL models by FG: Bi=initial estimated biomass, P/B=production/biomass, Q/B=consumption/biomass, Landing and Discard. The asterisks indicate biomass estimated for the FG fixing the Ecotrophic Efficiency= 0.90 (*), 0.95 (**) and 0.99 (**). Input data changed during the model balancing are in bold.

| FG | Bi (t km^-2^) | | P/B (year^-1^) | | Q/B (year^-1^) | | Landing (t km^-2^ year^-1^) | | Discard (t km^-2^ year^-1^) | |
| --- | --- | --- | --- | --- | --- | --- | --- | --- | --- | --- |
|  | CAL | SAL | CAL | SAL | CAL | SAL | CAL | SAL | CAL | SAL |
| 1 | 0.0170 | 0.0335 | 0.064 | 0.064 | 18.760 | 18.760 |  |  |  |  |
| 2 | 0.0064 | 0.0123 | 0.040 | 0.040 | 4.110 | 4.110 |  |  |  |  |
| 3 | 0.0038 | 0.0074 | 0.270 | 0.270 | 3.500 | 3.500 |  |  | 0.00099 | 0.00015 |
| 4 | 0.0008 | 0.0012 | 0.140 | 0.140 | 60.830 | 60.830 |  |  | 0.00002 | 0.00003 |
| 5 | **0.0310** | **0.0257** | **0.582** | **0.650** | **5.000** | **5.000** | 0.01613 | 0.01352 | 0.00055 | 0.00009 |
| 6 | 0.0110 | **0.0186** | **0.514** | 0.593 | **2.095** | **3.080** |  |  | 0.00005 | 0.00520 |
| 7 | 0.0058 | 0.0016 | 0.665 | 0.600 | 3.150 | 3.400 | 0.00006 | 0.00003 | 0.00000 | 0.00076 |
| 8 | **0.0638** | 0.0404 | **0.754** | 0.785 | 3.600 | 3.600 | 0.00004 | 0.00564 | 0.02743 | 0.00286 |
| 9 | 0.0569 | 0.0284 | 0.520 | 0.675 | **3.232** | **4.293** |  |  | 0.00713 | 0.01247 |
| 10 | **0.0203** | 0.0122 | 0.650 | 0.650 | **4.200** | **4.200** |  |  | 0.00857 | 0.00257 |
| 11 | 0.0332 | 0.0321 | **0.444** | **0.450** | **2.473** | **2.473** | 0.00426 | 0.00426 | 0.00421 | 0.00155 |
| 12 | 0.0550 | **0.0401** | **1.035** | 1.035 | 4.307 | **4.198** | 0.02119 | 0.00796 | 0.00753 | 0.00592 |
| 13 | **0.1128** | **0.0713** | **1.186** | **0.951** | **4.935** | **4.182** | 0.00793 | 0.01371 | 0.01756 | 0.00556 |
| 14 | **0.1082** | **0.0533** | **1.318** | **1.318** | **7.280** | **6.587** | 0.10094 | 0.00305 | 0.00049 | 0.04297 |
| 15 | 0.1283 | **0.1219** | **2.548** | **3.327** | **10.794** | **12.852** |  |  | 0.03305 | 0.00327 |
| 16 | 0.0972 | 0.0751 | **1.139** | 1.179 | **4.544** | **4.544** | 0.00096 | 0.00663 | 0.07135 | 0.01742 |
| 17 | 0.3460 | **0.3650** | 1.296 | **0.840** | 5.739 | **5.056** |  |  | 0.00605 | 0.00017 |
| 18 | **0.3662** | **0.4220** | **0.699** | **0.689** | **2.923** | **2.888** | 0.04233 | 0.00538 | 0.07357 | 0.01045 |
| 19 | 0.4451 | **0.2863** | **0.991** | **1.008** | **5.711** | **6.085** | 0.07222 | 0.10536 | 0.06493 | 0.00425 |
| 20 | **0.4087** | **0.2880** | **1.055** | **1.055** | 4.898 | **5.803** | 0.01214 | 0.05635 | 0.00372 | 0.00284 |
| 21 | **0.0211** | 0.0333 | **0.560** | **0.560** | **2.500** | 2.500 |  |  | 0.00005 | 0.00002 |
| 22 | 0.5778 | **0.3597** | **1.221** | **1.220** | **7.766** | **7.174** |  |  | 0.04855 | 0.00577 |
| 23 | **1.0190** | **0.9251** | **1.370** | **1.496** | **9.533** | **10.653** | 0.03412 | 0.01390 | 0.00931 | 0.00385 |
| 24 | **0.3034** | **0.2141** | **1.231** | **1.391** | **5.912** | **5.912** | 0.07025 | 0.07241 | 0.00136 | 0.00084 |
| 25 | **0.4741** | **0.2521** | **1.038** | **0.916** | **5.288** | **4.894** |  |  | 0.02502 | 0.00146 |
| 26 | **0.5272** | **0.6044** | **3.000** | **3.000** | **10.777** | **11.010** |  |  | 0.00032 | 0.00096 |
| 27 | 0.0561 | **0.0356** | 1.190 | 1.190 | **5.660** | **5.660** | 0.02323 | 0.02316 | 0.00001 | 0.00001 |
| 28 | 0.0413 | 0.0500 | **1.073** | **1.410** | 6.640 | 8.614 | 0.02677 | 0.04408 | 0.00002 |  |
| 29 | 0.0078 | 0.0144 | **0.869** | **0.846** | 4.030 | 4.030 | 0.00635 | 0.00928 | 0.00000 | 0.00001 |
| 30 | 0.0537 | 0.0441 | 1.110 | 1.110 | 4.500 | 4.500 |  |  | 0.00543 | 0.00038 |
| 31 | 0.0482 | **0.0225** | 2.176 | **2.227** | **11.937** | **12.195** |  | 0.02195 | 0.00401 | 0.00110 |
| 32 | **0.0559** | **0.0588** | **2.940** | **2.940** | **16.112** | **16.112** | 0.00409 | 0.00193 | 0.00147 | 0.00134 |
| 33 | 0.1041 | **0.1505** | **3.967** | **2.221** | **14.628** | **15.903** | 0.10746 | 0.03288 | 0.00071 | 0.00052 |
| 34 | **0.0293** | **0.0353** | 3.361 | **3.380** | **13.159** | **12.422** |  |  | 0.00111 | 0.00019 |
| 35 | 0.0338 | 0.0597 | **4.812** | **3.670** | 18.235 | **18.245** |  |  | 0.00005 | 0.00073 |
| 36 | **0.7066** | **0.8340** | **3.175** | **3.222** | **12.771** | **11.635** |  |  | 0.01686 | 0.00323 |
| 37 | **0.3345** | 0.2735 | **1.054** | **1.226** | **5.977** | **5.634** | 0.01160 | 0.02517 | 0.00576 | 0.00264 |
| 38 | **0.2283** | 0.1728 | **2.324** | 2.333 | **10.296** | **10.335** |  |  | 0.00062 | 0.00054 |
| 39 | **0.4665** | **0.3200** | 2.690 | **2.845** | 11.780 | **11.437** |  |  | 0.00130 | 0.00031 |
| 40 | 0.8680 | **0.3129** | **1.674** | **2.319** | 10.932 | **10.835** | 0.07011 | 0.01454 | 0.02986 | 0.00195 |
| 41 | 0.2084 | 0.1342 | 1.414 | **1.414** | 8.867 | **8.867** | 0.19338 | 0.10486 | 0.00004 | 0.00005 |
| 42 | 0.0617 | 0.0447 | 1.165 | 1.165 | 7.318 | 7.318 | 0.04345 | 0.02860 |  | 0.00001 |
| 43 | 0.1019 | 0.0937 | **0.925** | **0.726** | 7.318 | 7.318 | 0.05924 | 0.04992 |  | 0.00050 |
| 44 | 0.1165 | 0.1017 | 2.741 | 2.741 | 12.571 | 12.571 |  |  | 0.00482 | 0.00151 |
| 45 | 1.974* | 1.066* | 5.140 | 5.140 | 20.708 | 20.708 |  |  |  |  |
| 46 | 2.838* | 1.497* | 5.306 | 5.306 | 22.536 | 22.536 |  |  | 0.01339 | 0.00853 |
| 47 | 0.030** | 0.035** | 13.570 | 13.570 | 48.800 | 48.800 |  |  |  |  |
| 48 | 1.535** | 1.360** | 7.730 | 7.730 | 36.510 | 36.510 |  |  |  |  |
| 49 | 1.510*** | 1.324*** | 18.000 | 18.000 | 66.000 | 66.000 |  |  |  |  |

(continued overleaf)

Table S2. Input parameters of the CAL and SAL models by FG: Bi=initial estimated biomass, P/B=production/biomass, Q/B=consumption/biomass, Landing and Discard. The asterisks indicate biomass estimated for the FG fixing the Ecotrophic Efficiency=0.90 (*), 0.95(**) and 0.99 (***). Input data changed during the model balancing are in bold.

| FG | Bi (t km^-2^) | | P/B (year^-1^) | | Q/B (year^-1^) | | Landing (t km^-2^ year^-1^) | | Discard (t km^-2^ year^-1^) | |
| --- | --- | --- | --- | --- | --- | --- | --- | --- | --- | --- |
|  | CAL | SAL | CAL | SAL | CAL | SAL | CAL | SAL | CAL | SAL |
| 50 | 2.255*** | 1.859*** | 28.550 | 28.550 | 107.460 | 107.460 |  |  |  |  |
| 51 | 3.7003 | 3.7003 | 20.870 | 20.870 | 80.730 | 80.730 |  |  |  |  |
| 52 | 6.0000 | 6.0000 | 25.870 | 25.870 | 89.780 | 89.780 |  |  |  |  |
| 53 | 0.5100 | 0.5900 | 5.240 | 5.240 |  |  |  |  |  |  |
| 54 | 3.0683 | 3.0683 | 175.785 | 175.785 |  |  |  |  |  |  |
| 55 | 2.8936 | 2.8936 | 205.620 | 205.620 |  |  |  |  |  |  |
| 56 | 1.0000 | 1.0000 |  |  |  |  |  |  |  |  |
| 57 | 1.0000 | 1.0000 |  |  |  |  |  |  |  |  |
| 58 | 1.9400 | 1.6900 |  |  |  |  |  |  |  |  |

Table S3. Main ecological indices obtained by the Ecopath model in the CAL and SAL food webs. Trophic Level (TL), Ecotrophic Efficiency (EE), Production (P/Q), Omnivory Index (OI), Fishing Mortality (F) and Keystoness index (KSi).

| N | TL | | EE | | P/Q | | OI | | F | | KS | |
| --- | --- | --- | --- | --- | --- | --- | --- | --- | --- | --- | --- | --- |
|  | CAL | SAL | CAL | SAL | CAL | SAL | CAL | SAL | CAL | SAL | CAL | SAL |
| 1 | 4.86 | 4.72 | 0.000 | 0.000 | 0.003 | 0.003 | 0.46 | 0.43 |  |  | -0.29 | -0.11 |
| 2 | 4.05 | 4.04 | 0.000 | 0.000 | 0.010 | 0.010 | 1.31 | 1.05 |  |  | -2.25 | -1.97 |
| 3 | 3.55 | 3.63 | 0.962 | 0.076 | 0.077 | 0.077 | 0.85 | 0.84 | 0.26 | 0.02 | -1.54 | -2.15 |
| 4 | 3.45 | 3.71 | 0.191 | 0.181 | 0.002 | 0.002 | 1.28 | 1.32 | 0.03 | 0.03 | -1.89 | -1.66 |
| 5 | 5.23 | 5.00 | 0.925 | 0.815 | 0.116 | 0.130 | 1.66 | 1.50 | 0.54 | 0.53 | -0.35 | -0.70 |
| 6 | 4.94 | 4.93 | 0.009 | 0.472 | 0.245 | 0.193 | 0.28 | 0.32 | 0.00 | 0.28 | -1.10 | -0.51 |
| 7 | 4.57 | 4.57 | 0.145 | 0.863 | 0.211 | 0.176 | 0.11 | 0.24 | 0.01 | 0.49 | -1.69 | -2.06 |
| 8 | 4.49 | 4.39 | 0.687 | 0.279 | 0.209 | 0.218 | 0.17 | 0.43 | 0.43 | 0.21 | -0.74 | -0.90 |
| 9 | 4.27 | 4.33 | 0.533 | 0.852 | 0.161 | 0.157 | 0.75 | 0.82 | 0.13 | 0.44 | -0.17 | -0.60 |
| 10 | 4.56 | 4.55 | 0.940 | 0.733 | 0.155 | 0.155 | 0.19 | 0.15 | 0.42 | 0.21 | -0.95 | -1.17 |
| 11 | 4.78 | 4.79 | 0.686 | 0.462 | 0.180 | 0.182 | 0.18 | 0.15 | 0.26 | 0.18 | -0.58 | -0.68 |
| 12 | 3.94 | 3.95 | 0.844 | 0.668 | 0.240 | 0.247 | 0.58 | 0.61 | 0.52 | 0.35 | -0.96 | -0.85 |
| 13 | 4.11 | 4.06 | 0.564 | 0.830 | 0.240 | 0.227 | 0.55 | 0.54 | 0.23 | 0.27 | -0.52 | -0.93 |
| 14 | 4.31 | 4.26 | 0.858 | 0.959 | 0.181 | 0.200 | 0.35 | 0.35 | 0.94 | 0.86 | -0.33 | -0.68 |
| 15 | 4.06 | 4.15 | 0.785 | 0.496 | 0.236 | 0.259 | 0.51 | 0.41 | 0.26 | 0.03 | -0.28 | -0.26 |
| 16 | 4.20 | 4.27 | 0.845 | 0.932 | 0.251 | 0.259 | 0.24 | 0.25 | 0.74 | 0.32 | -0.67 | -0.76 |
| 17 | 3.64 | 3.68 | 0.432 | 0.566 | 0.226 | 0.166 | 0.19 | 0.17 | 0.02 | 0.00 | -0.61 | -0.40 |
| 18 | 3.80 | 3.86 | 0.928 | 0.781 | 0.239 | 0.238 | 0.33 | 0.35 | 0.32 | 0.04 | -0.30 | -0.38 |
| 19 | 3.67 | 3.74 | 0.858 | 0.872 | 0.173 | 0.166 | 0.14 | 0.22 | 0.31 | 0.38 | -0.35 | -0.33 |
| 20 | 3.53 | 3.70 | 0.501 | 0.530 | 0.215 | 0.182 | 0.34 | 0.47 | 0.04 | 0.21 | -0.74 | -0.47 |
| 21 | 4.10 | 4.01 | 0.611 | 0.258 | 0.224 | 0.224 | 0.22 | 0.53 | 0.00 | 0.00 | -1.43 | -0.99 |
| 22 | 3.54 | 3.55 | 0.838 | 0.574 | 0.157 | 0.170 | 0.08 | 0.08 | 0.08 | 0.02 | -0.53 | -0.66 |
| 23 | 3.39 | 3.41 | 0.914 | 0.973 | 0.144 | 0.140 | 0.06 | 0.06 | 0.04 | 0.02 | -0.24 | -0.25 |
| 24 | 4.07 | 4.09 | 0.904 | 0.880 | 0.208 | 0.235 | 0.28 | 0.42 | 0.24 | 0.34 | -0.14 | -0.07 |
| 25 | 3.85 | 3.97 | 0.742 | 0.805 | 0.196 | 0.187 | 0.26 | 0.26 | 0.05 | 0.01 | -0.39 | -0.45 |
| 26 | 3.48 | 3.50 | 0.931 | 0.896 | 0.278 | 0.272 | 0.29 | 0.31 | 0.00 | 0.00 | -0.54 | -0.47 |
| 27 | 3.80 | 3.85 | 0.853 | 0.861 | 0.210 | 0.210 | 0.11 | 0.09 | 0.41 | 0.65 | -1.32 | -1.15 |
| 28 | 4.51 | 4.61 | 0.966 | 0.915 | 0.125 | 0.164 | 0.12 | 0.12 | 0.65 | 0.88 | -0.65 | -0.41 |
| 29 | 4.88 | 4.94 | 0.939 | 0.762 | 0.216 | 0.210 | 0.11 | 0.12 | 0.82 | 0.64 | -0.11 | -0.67 |
| 30 | 3.68 | 3.72 | 0.944 | 0.251 | 0.247 | 0.247 | 0.05 | 0.20 | 0.10 | 0.01 | -1.12 | -0.98 |
| 31 | 4.76 | 4.87 | 0.694 | 0.962 | 0.182 | 0.183 | 0.18 | 0.15 | 0.08 | 1.02 | -0.10 | -0.26 |
| 32 | 4.42 | 4.40 | 0.818 | 0.868 | 0.182 | 0.182 | 0.20 | 0.18 | 0.10 | 0.06 | -0.36 | -0.28 |
| 33 | 3.90 | 3.81 | 0.862 | 0.937 | 0.271 | 0.140 | 0.29 | 0.37 | 1.04 | 0.22 | -0.40 | -0.25 |
| 34 | 4.18 | 4.24 | 0.811 | 0.806 | 0.255 | 0.272 | 0.57 | 0.42 | 0.04 | 0.01 | -0.60 | -0.32 |
| 35 | 3.86 | 3.87 | 0.748 | 0.707 | 0.264 | 0.201 | 0.02 | 0.02 | 0.00 | 0.01 | -1.07 | -0.86 |
| 36 | 3.43 | 3.44 | 0.864 | 0.776 | 0.249 | 0.277 | 0.49 | 0.49 | 0.02 | 0.00 | -0.21 | -0.22 |
| 37 | 3.27 | 3.38 | 0.745 | 0.834 | 0.176 | 0.218 | 0.54 | 0.55 | 0.05 | 0.10 | -0.88 | -0.56 |
| 38 | 3.41 | 3.47 | 0.673 | 0.950 | 0.226 | 0.226 | 0.28 | 0.34 | 0.00 | 0.00 | -0.49 | -0.50 |
| 39 | 3.23 | 3.18 | 0.531 | 0.900 | 0.228 | 0.249 | 0.39 | 0.52 | 0.00 | 0.00 | -0.49 | -0.31 |
| 40 | 3.38 | 3.40 | 0.796 | 0.800 | 0.153 | 0.214 | 0.29 | 0.35 | 0.12 | 0.05 | -0.36 | -0.48 |
| 41 | 3.50 | 3.60 | 0.920 | 0.952 | 0.159 | 0.159 | 0.46 | 0.52 | 0.83 | 0.78 | -0.41 | -0.41 |
| 42 | 3.96 | 4.04 | 0.892 | 0.837 | 0.159 | 0.159 | 0.26 | 0.22 | 0.70 | 0.64 | -0.64 | -0.75 |
| 43 | 3.47 | 3.49 | 0.876 | 0.903 | 0.126 | 0.099 | 0.52 | 0.47 | 0.58 | 0.54 | -0.81 | -0.74 |
| 44 | 3.62 | 3.56 | 0.972 | 0.878 | 0.218 | 0.218 | 0.57 | 0.53 | 0.04 | 0.01 | -0.72 | -0.84 |
| 45 | 2.71 | 2.73 | 0.900 | 0.900 | 0.248 | 0.248 | 0.50 | 0.51 |  |  | -0.20 | -0.22 |
| 46 | 2.41 | 2.59 | 0.900 | 0.900 | 0.235 | 0.235 | 0.38 | 0.54 |  |  | -0.12 | -0.16 |
| 47 | 2.96 | 2.98 | 0.950 | 0.950 | 0.278 | 0.278 | 0.54 | 0.54 |  |  | -0.84 | -0.80 |
| 48 | 2.59 | 2.59 | 0.950 | 0.950 | 0.212 | 0.212 | 0.55 | 0.55 |  |  | -0.29 | -0.32 |
| 49 | 2.88 | 2.88 | 0.990 | 0.990 | 0.273 | 0.273 | 0.34 | 0.35 |  |  | 0.00 | 0.01 |

(continued overleaf)

Table S3. Main ecological indices obtained by the Ecopath model in the CAL and SAL food webs. Trophic Level (TL), Ecotrophic Efficiency (EE), Production (P/Q), Omnivory Index (OI), Fishing Mortality (F) and Keystoness index (KSi).

| N | TL | EE | P/Q | OI | F | KS | TL | EE | P/Q | OI | F | KS |
| --- | --- | --- | --- | --- | --- | --- | --- | --- | --- | --- | --- | --- |
|  | CAL | SAL | CAL | SAL | CAL | SAL | CAL | SAL | CAL | SAL | CAL | SAL |
| 50 | 2.28 | 2.30 | 0.990 | 0.990 | 0.266 | 0.266 | 0.27 | 0.28 |  |  | -0.11 | -0.10 |
| 51 | 2.32 | 2.32 | 0.980 | 0.931 | 0.259 | 0.259 | 0.22 | 0.22 |  |  | -0.20 | -0.18 |
| 52 | 2.00 | 2.00 | 0.927 | 0.855 | 0.288 | 0.288 |  |  |  |  | -0.93 | -0.95 |
| 53 | 1.00 | 1.00 | 0.583 | 0.379 |  |  |  |  |  |  | -1.05 | -0.84 |
| 54 | 1.00 | 1.00 | 0.304 | 0.280 |  |  |  |  |  |  | -0.52 | -0.52 |
| 55 | 1.00 | 1.00 | 0.398 | 0.360 |  |  |  |  |  |  | -0.30 | -0.30 |
| 56 | 1.00 | 1.00 | 0.513 | 0.494 |  |  |  |  |  |  |  |  |
| 57 | 1.00 | 1.00 | 0.267 | 0.905 |  |  |  |  |  |  |  |  |
| 58 | 1.00 | 1.00 | 0.124 | 0.086 |  |  |  |  |  |  |  |  |
