# Supplementary material for: Ecosystem functioning of two marine food webs in the North‐Western Ionian Sea (Central Mediterranean Sea)
Source: Ecol Evol. 2019 Sep 3;9(18):10198–212. doi: 10.1002/ece3.5527 (PMC6787816; doi:10.1002/ece3.5527)
Supplement: Supplementary file 2 [file ECE3-9-10198-s002.docx]

**Appendix S1**

**The Reiterative Aggregation Method based on trophic similarity and bathymetric distribution**

The Reiterative Aggregation Method used in the building of the benthopelagic and demersal FGs combined the trophic similarity and the bathymetric distribution of species. A bathymetric distribution indicator of the species biomass was the Centre Of Gravity (COG, Daget, 1976), a synthetic measure that indicates the depth to which a species shows the highest biomass concentration, with a value of variance indicating the displacement of biomass species between bathymetric layers and it is expressed as:

$$COG={(X-1+2X_{2}+3X_{3}+4X_{4}+\ldots+\mathrm{nX}_{n})}/{\sum X\_i}$$

where X represents the value of the average biomass of the species in the layer i. In particular, 8 bathymetric layers of 100 m have been identified between 10 and 800 m depth.

The quantitative information on diet preferences (in weight) have been collected for 129 species on a total of 276 species sampled in the benthopelagic and demersal assemblages of study areas, through the scientific published and gray literature both of local and nearby geographical areas. The analysis of diet data has been carried out by implementing a bi-clustering on the matrix of prey-predator relationships, also using the vector of weighting factor [COG], implemented by means of a Microsoft Visual Basic routine. The rectangular matrix of prey-predator relationships [X] has dimensions N (number of preys) x M number of predators, with N>M. That is to say that N = M+ M’ where the M’ are the species for which no diet is available and compare only as preys. Each element of the matric xij represents the proportion (expressed as %) in wet weight preferably of the prey i in the diet of the predator j, thus:

$\mathbf{x}_{\mathbf{.j}}\mathbf{=}\sum_{\mathbf{i=1}}^{\mathbf{N}} \mathbf{x}_{\mathbf{ij}}\mathbf{=100}$ (1)

The vector [G] enables the assignment of each species to a functional group. Given that the assignment of a species to a functional groups is here based only on diet specifications, the vector [G] has dimension 1xM, where elements gj can have similar value to specify the belonging to the same functional group. By opportunely and freely choosing values of this vector it is possible to define k groups (Gk) with 1<k<M.

Given a vector of groups identification [G] the matrix [X] is changed into new diet matrix [Y*] that account for the aggregation of the species. Aggregation matrix [Y*] is built through a two-steps process, by first accounting aggregation by columns (predators), i.e. elements of the transitional matrix [Y’] are:

${\mathbf{y}\mathbf{'}}_{\mathbf{ij}}\mathbf{=}\frac{\sum_{\mathbf{gj}\boldsymbol{\in}\mathbf{Gk}} \mathbf{x}_{\mathbf{ij}}}{\sum_{\mathbf{gj}\boldsymbol{\in}\mathbf{Gk}} \mathbf{1}}$ (2)

Of course, if only an element pertain to the group Gk, then y’ij = xij. IN this way the predators species pertaining to the same group have the same diet, equal to the average of the diets of the species in the group. This allow conservation of dimension, so matrix [Y’] has same dimensions of original [X]. The loss of information due to trophic aggregation of the first step was evaluated by calculating the sum of squared difference between elements of matrixes [X] and [Y’]:

$\boldsymbol{\theta}^{\mathbf{0}}\mathbf{=}\sum_{\mathbf{i}\mathbf{=}\mathbf{1}}^{\mathbf{N}} \sum_{\mathbf{j}\mathbf{=}\mathbf{1}}^{\mathbf{M}} \left( \frac{{\mathbf{Y}\mathbf{'}}_{\mathbf{ij}}}{\mathbf{100}}\mathbf{-}\frac{\mathbf{X}_{\mathbf{ij}}}{\mathbf{100}} \right)^{\mathbf{2}}$ (3)

This first step can be considered similar to a cluster analysis performed using Euclidean distance and average linkage clustering. However, being some predators also prey for other species, the aggregation of the M predators in k groups affect also the preys aggregation. Thus the final matrix [Y*] accounts for this by performing a second step 2 in which, the accounting of aggregation is done in similar way as in step 1 but by rows. Thus elements of the final matrix [Y*] are:

$\boldsymbol{y}_{\boldsymbol{ij}}^{\boldsymbol{*}}\boldsymbol{=}\frac{\sum_{\mathbf{gi}\boldsymbol{\in}\mathbf{Gk}} {\mathbf{y}\mathbf{'}}_{\mathbf{ij}}}{\sum_{\mathbf{gi}\boldsymbol{\in}\mathbf{Gk}} \mathbf{1}} \mathbf{for} \mathbf{any} \mathbf{i}\boldsymbol{\leq}\mathbf{j}$ (4)

$\mathbf{y}_{\mathbf{ij}}^{\mathbf{*}}\mathbf{=}{\mathbf{y}\mathbf{'}}_{\mathbf{ij}}\mathbf{for} \mathbf{any} \mathbf{i}>j$ (5)

The final matrix [Y*] represent the new prey predator matrix, based on [X] and accounting of aggregation indicated in [G]. Other than predators, also preys that are included in the same group have identical predatory losses terms y*ij , equal to the average of the predatory terms of the species in the group. This allow conservation of dimension, so matrix [Y*] has same dimensions of original [X]. The loss of information due to trophic aggregation was evaluated by calculating the sum of squared difference between elements of matrixes [X] and [Y*]:

$\boldsymbol{\theta}\mathbf{=}\sum_{\mathbf{i}\mathbf{=}\mathbf{1}}^{\mathbf{N}} \sum_{\mathbf{j}\mathbf{=}\mathbf{1}}^{\mathbf{M}} \left( \frac{{\mathbf{y}\mathbf{*}}_{\mathbf{ij}}}{\mathbf{100}}\mathbf{-}\frac{\mathbf{X}_{\mathbf{ij}}}{\mathbf{100}} \right)^{\mathbf{2}}$ (6)

In order to increase the importance of small proportions in the loss function an additional form was used:

$\boldsymbol{ln\theta}\mathbf{=}\sum_{\mathbf{i}\mathbf{=}\mathbf{1}}^{\mathbf{N}} \sum_{\mathbf{j}\mathbf{=}\mathbf{1}}^{\mathbf{M}} \left( \mathbf{ln}\left( {\mathbf{y}\mathbf{*}}_{\mathbf{ij}}\mathbf{+}\mathbf{1} \right)\mathbf{-}\mathbf{ln}\mathbf{(}\mathbf{x}_{\mathbf{ij}}\mathbf{+}\mathbf{1}\mathbf{)} \right)^{\mathbf{2}}$ (6b)

Further, the aggregation can account for the preferred habitat of species, by using the COG information for weighting the distances. The vectors [P] and [S] are the average preferred depth of occurrence of prey species (named COG), and are calculated from trawl surveys monitoring. These vectors has dimension N and pi and si specify the preferred depth and variance for the preys. Similarly vectors [P’] and [S’] of dimension M define the preferred depth and variance (COG and its variance) for the M predators. From these information were built different matrixes of weighting factors. [A] corresponds to the matrix of absolute differences of preferred depths, whose elements are:

$\mathbf{a}_{\mathbf{ij}}\mathbf{=}\left| \mathbf{p}_{\mathbf{i}}\mathbf{-}{\mathbf{p}\mathbf{'}}_{\mathbf{j}} \right|$ (7)

[T] corresponds to a distance similar to that of t-student for comparing two means with different variances:

$\mathbf{t}_{\mathbf{ij}}\mathbf{=}\frac{\left| \mathbf{p}_{\mathbf{i}}\mathbf{-}{\mathbf{p}\mathbf{'}}_{\mathbf{j}} \right|}{\sqrt{\mathbf{s}_{\mathbf{i}}\mathbf{+}{\mathbf{s}\mathbf{'}}_{\mathbf{j}}}}$ (8)

Finally [B] corresponds to the Bhattacharyya distance between two normal distributions:

$\mathbf{B}_{\mathbf{ij}}\mathbf{=}\frac{\mathbf{1}}{\mathbf{4}}\mathbf{ln}\left[ \frac{\mathbf{1}}{\mathbf{4}}\left( \frac{\mathbf{s}_{\mathbf{i}}}{\mathbf{s}_{\mathbf{j}}}\mathbf{+}\frac{\mathbf{s}_{\mathbf{j}}}{\mathbf{s}_{\mathbf{i}}}\mathbf{+}\mathbf{2} \right) \right]\mathbf{+}\frac{\mathbf{1}}{\mathbf{4}}\left( \frac{\left( \mathbf{p}_{\mathbf{i}}\mathbf{-}{\mathbf{p}\mathbf{'}}_{\mathbf{j}} \right)^{\mathbf{2}}}{\mathbf{s}_{\mathbf{i}}\mathbf{+}{\mathbf{s}\mathbf{'}}_{\mathbf{j}}} \right)$ (9)

Each weighting matrix was scaled to the maximum values to obtain normalized weights (Legendre & Legendre, 2012) and thus obtaining ([A]) ̃ ([T]) ̃ ([B]) ̃. Using the three weighting systems we could calculate the weighted loss functions also accounting for distances of preferred depths:

$\boldsymbol{\theta'=}\sum_{\mathbf{i=}\mathbf{1}}^{\mathbf{N}} \sum_{\mathbf{j=}\mathbf{1}}^{\mathbf{M}} {{\tilde{\mathbf{a}}}_{\mathbf{ij}}\left( \frac{\mathbf{y*}_{\mathbf{ij}}}{\mathbf{100}}\mathbf{-}\frac{\mathbf{x}_{\mathbf{ij}}}{\mathbf{100}} \right)}^{\mathbf{2}}$ (10)

$\boldsymbol{\theta''=}\sum_{\mathbf{i=}\mathbf{1}}^{\mathbf{N}} \sum_{\mathbf{j=}\mathbf{1}}^{\mathbf{M}} {{\tilde{\mathbf{t}}}_{\mathbf{ij}}\left( \frac{\mathbf{y*}_{\mathbf{ij}}}{\mathbf{100}}\mathbf{-}\frac{\mathbf{x}_{\mathbf{ij}}}{\mathbf{100}} \right)}^{\mathbf{2}}$ (11)

$\boldsymbol{\theta'''=}\sum_{\mathbf{i=}\mathbf{1}}^{\mathbf{N}} \sum_{\mathbf{j=}\mathbf{1}}^{\mathbf{M}} {{\tilde{\mathbf{b}}}_{\mathbf{ij}}\left( \frac{\mathbf{y*}_{\mathbf{ij}}}{\mathbf{100}}\mathbf{-}\frac{\mathbf{x}_{\mathbf{ij}}}{\mathbf{100}} \right)}^{\mathbf{2}}$ (12)

By changing [G] different final aggregation matrixes [Y*] can be built and performances evaluated using the different loss functions.

In order to evaluate the aggregation effect and the loss of trophic information by different grouping was implemented a reiterative random method by choosing r = 20000 random grouping with k ranging from 4 to 130. The r runs were evaluated in terms of loss functions calculated above also considering the different weighting factors.

**References:**

Daget, P. 1976. Ordinations des profile ecologiques. Natural Monspel., Ser. Biolog., 26, 109-128.

Legendre, P., and Legendre, L.. 2012. Numerical ecology, 3rd edn. Elsevier Science BV, Amsterdam

Figure S1: Calabria Diet Matrix.

Figure S2: Salento Diet Matrix.

**PREBAL Analysis**

**
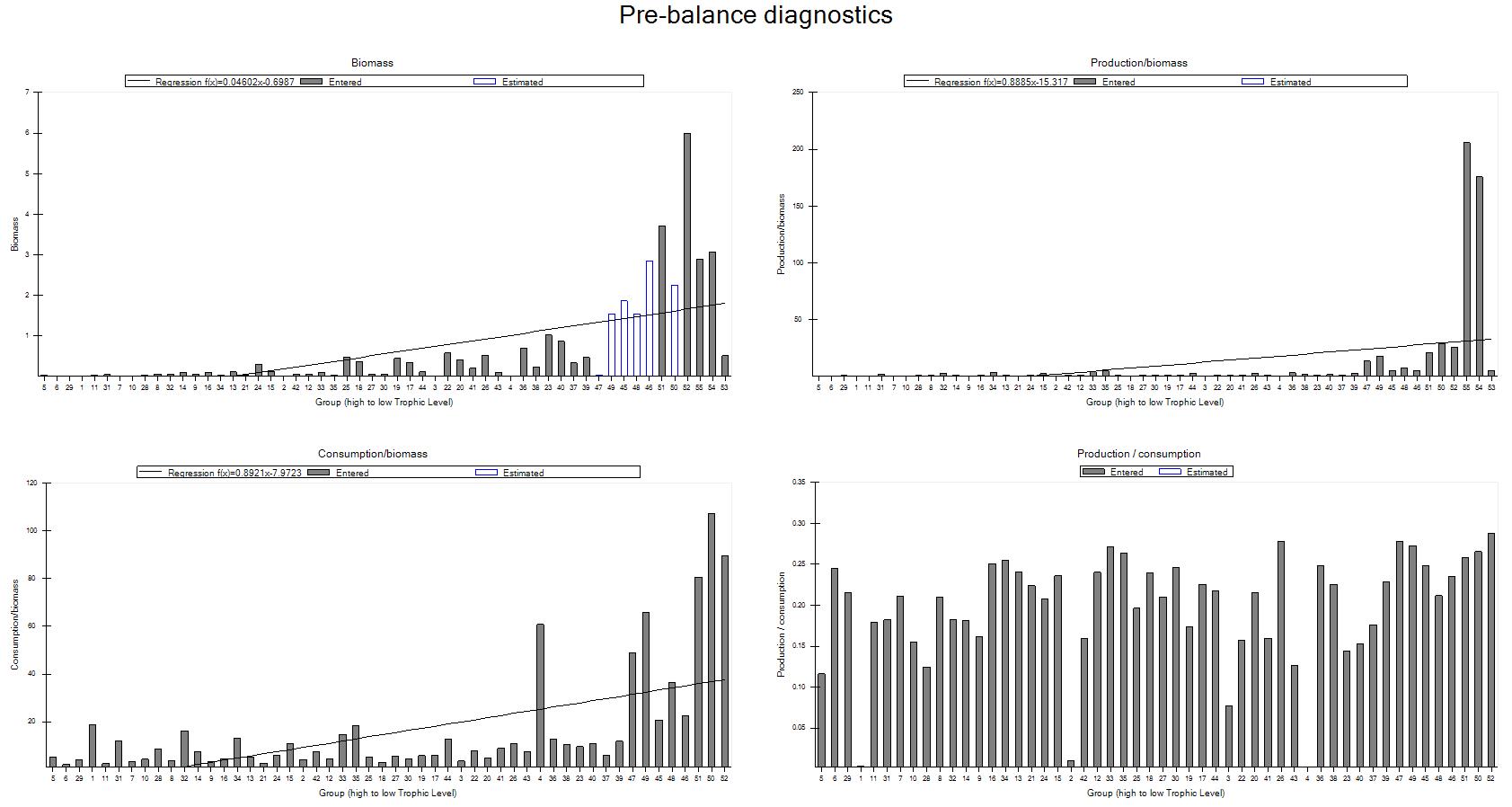
**

Figure S3: PREBAL diagnostics depicting values obtained following the manual mass-balance procedure of the CAL model. TL increase from right to left. White bars indicate data estimated by the model.

**
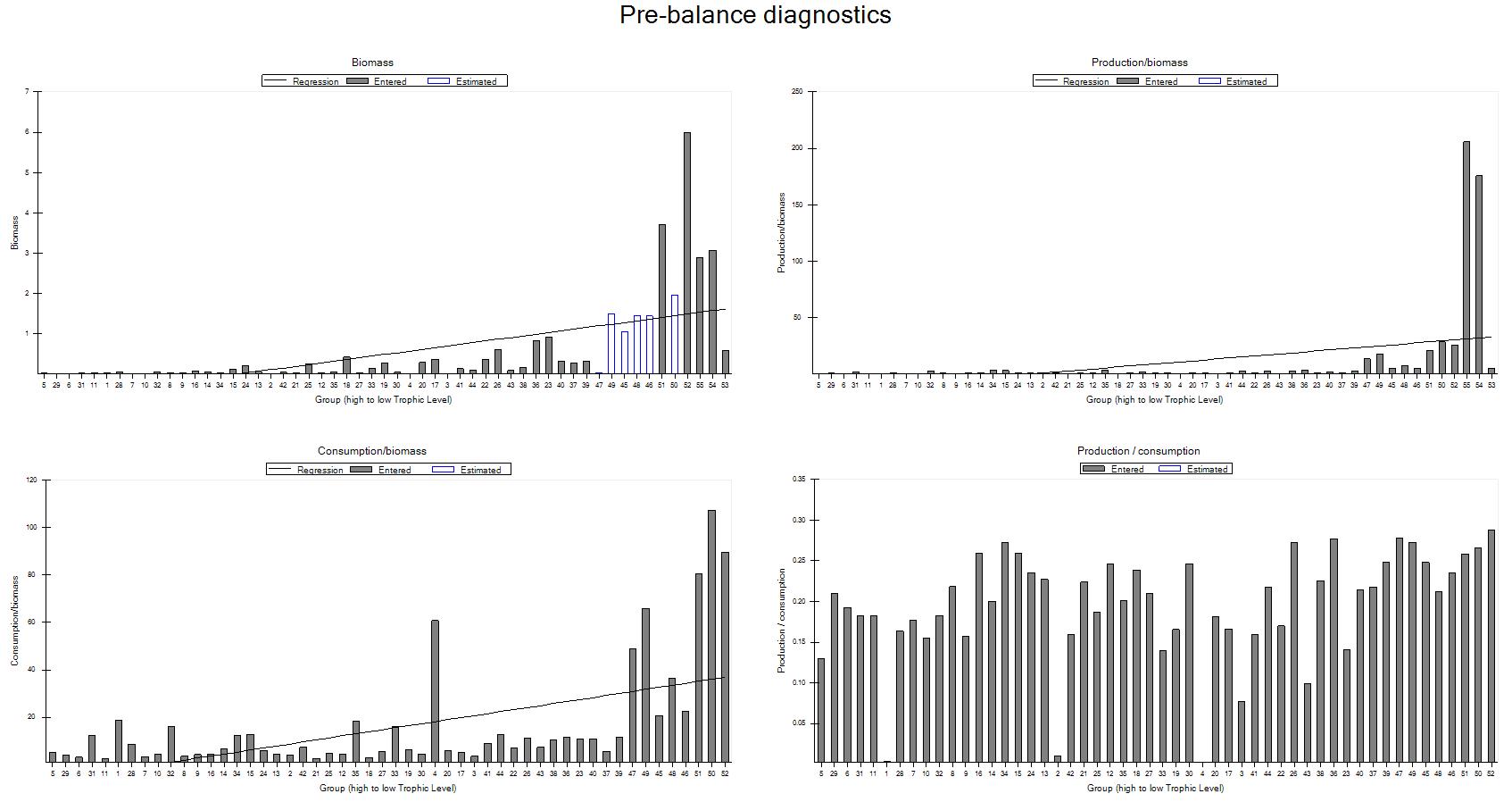
**

Figure S4: PREBAL diagnostics depicting values obtained following the manual mass-balance procedure of the SAL model. TL increase from right to left. Wihte bars indicate data estimated by the model.
